# Traditional Chinese Patent Medicine for Acute Ischemic Stroke: An Overview of Systematic Reviews Based on the GRADE Approach

**DOI:** 10.1097/MD.0000000000002986

**Published:** 2016-03-25

**Authors:** Xin Zhang, Xue-Ting Liu, De-Ying Kang

**Affiliations:** From the Department of Integrated Traditional Chinese and Western Medicine(XZ); and Department of Evidence-Based Medicine and Clinical Epidemiology (X-TL, D-YK), West China Hospital, Sichuan University, Chengdu, China.

## Abstract

The aim of the study is to conduct an overview of systematic reviews (SRs) to provide a contemporary review of the evidence for delivery of Traditional Chinese Patent Medicine (TCPMs) for patients with acute ischemic stroke.

SRs were assessed for quality using the Assessment of Multiple Systematic Reviews (AMSTAR) tool and the Oxman-Guyatt Overview Quality Assessment Questionnaire (OQAQ). We assessed the quality of the evidence of high methodological quality (an AMSTAR score ≥9 or an OQAQ score ≥7) for reported outcomes using the GRADE (the Grading of Recommendations Assessment, Development and Evaluation) approach.

(1) Dan Shen agents: tiny trends toward the improvement in different neurological outcomes (RR = 1.16, 1.10, 1.23, 1.08, 1.12); (2) Mailuoning: a tiny trend toward improvement in the neurological outcome (RR = 1.18); (3) Ginkgo biloba: tiny trends toward improvement in the neurological outcome (RR = 1.18, MD = 0.81); (4) Dengzhanhua: a tiny trend toward an improvement in neurological (RR = 1.23); (5) Acanthopanax: a small positive (RR = 1.17, 1.31) result on neurological improvement reported; (6) Chuanxiong-type preparations: neurological functional improved (MD = 2.90);(7) Puerarin: no better effect on the rate of death or disability (OR = 0.81, 95% CI 0.35–1.87); (8) Milk vetch: no better effect on the rate of death (OR = 0.66, 95% CI: 0.11–2.83);(9) Qingkailing: rate of death reduced (OR = 0.66, 95% CI: 0.11–2.83). Limitations in the methodological quality of the RCTs, inconsistency and imprecision led to downgrading of the quality of the evidence, which varied by review and by outcome. Consequently, there are currently only weak evidences to support those TCPMs.

The 9 TCPMs may be effective in the treatment of acute ischemic stroke, as the GRADE approach indicated a weak recommendation for those TCPMs’ usage.

## INTRODUCTION

Stroke is the second most common cause of death in the world and the third most common cause of disability.^1,2^ Stroke is also the leading cause of death and long-term disability in China,^3,4^ where the rising incidence of stroke has created a serious public health problem.^5,6^ The most appreciable distinction between China and the West in treating stroke is the use of acupuncture and the traditional Chinese patent medicine (TCPM). The Chinese National Essential Drug (CNED) (2012 edition, available at http://www.sda.gov.cn), the Pharmacopoeia of the People's Republic of China (PPRC) (2010 edition), and the China Food and Drug Administration (CFDA, http://www.sda.gov.cn) list a variety of TCM preparations (mixtures of multi-herbs) subjected to a relatively strict drug evaluation process and widely used in current clinical practice for stroke in China. These TCM preparations are defined as TCPMs. TCPMs in CNED and PPRC represent the most important therapies listed by the China government, that may ameliorate the microcirculation are regularly used in acute ischemic stroke patients for >30 years. Nowadays, apart from aspirin and thrombolytic treatment with recombinant tissue plasminogen activator, no routine effective, generally accepted, specific treatment was applied for acute ischemic stroke. Assessment and confirmation of the effectiveness and safety of TCPMs, therefore, could have a significant impact on stroke management all over the world.

In the hierarchy of evidence-based medicine (EBM), systematic reviews (SRs) of high-quality randomized controlled trials (RCTs) are considered the best evidence regarding specific healthcare interventions.^7,8^ The portfolio of SRs has played an important role in informing evidence-base policy for Traditional Chinese Medicine (TCM) nationally and internationally.^5^ In spite of the availability of numerous publications regarding the use of TCPMs in stroke, the evidence from these SRs has not been evaluated systematically. Overview of SRs is designed to compile evidence from multiple SRs (addressing the effects of 2 or more potential interventions for a single condition or health problem) of interventions into 1 accessible and usable document, allowing the reader a quick overview of reviews relevant to a specific decision.^9^ Overviews can also help inform the strategic direction of conduct and structuring of future SRs and provide an opportunity to identify potential ’evidence gaps’. Typically, the Grading of Recommendations Assessment, Development and Evaluation (GRADE) approah^10^ is applied to rate the quality of a body of evidence in SRs and to determine the strength of recommendations during the overview process.^11–13^ The GRADE system clearly distinguishes between the quality of evidence and the strength of the recommendations and takes into account factors in addition to evidence to suggest appropriate therapeutic approaches,^11^ resulting in recommendations for or against an intervention based on whether the potential benefits of said intervention outweigh the potential harm caused or burden imposed by the intervention, as well as patient values and preferences.

To summarize evidence from >1 SR of different TCPMs in acute ischemic stroke, we conduct an overview in the first time that can be used by clinicians and policy makers in making decisions about the TCPMs.

## METHODS

This overview was conducted on the basis of the recommendations for Cochrane overviews.^14^ Ethical approval is not necessary for this overview research design.

### Inclusion Criteria for SRs

#### Types of Studies

Cochrane or non-Cochrane SRs of RCTs reporting outcomes with quantitative analyses were included. Data from original RCTs presented in >1 included SR were only analyzed once in this overview.

#### Study Participants

Ischemic stroke patients at acute stage (onset within 2 weeks) with any severity, either (1) diagnosed by brain magnetic resonance imaging (MRI) or brain computed tomography (CT) scan or (2) diagnosed corresponding to the World Health Organization definition,^15^ regardless of gender, age for severity of neurological deficit.

#### Types of interventions

TCPMs (mixtures of multi-herbs) listed in the PPRC (2010 edition), the CNED (2012 edition, available at http://www.sda.gov.cn), and approved by the China Food and Drug Administration (CFDA, http://www.sda.gov.cn) were included in our research. Either (1) comparison of a TCPM with placebo or routine treatment (2) or comparison of a TCPM plus routine treatments versus routine treatments alone was included.

#### Outcomes

A panel of 12 experts from our hospital specialized in neurosurgery, neurology, cardiovascular disease, and TCM was convened. This panel collected a priori as many important outcomes related to stroke as they can. Subsequently, they assessed numerically from 1 to 9 points to each outcome on the basis of their clinical importance (1 = least importance; 9 = most importance) and recorded their judgments privately. In general, these outcomes of patient interest, long-term direct outcomes, as well as specific measures related to TCPMs, were considered as important or crucial outcomes. We statistically aggregated the individual judgments to derive the median score of each outcome. Subsequently, the importance of each outcome was classified. On the basis of their importance regarding clinical decision making, outcomes were specified as 3 categories: limited importance (median score = 1 to 3), important but not critical (median score = 4 to 6) and critical (median score = 7 to 9)^10^ (Supplemental table I). The 2 categories of outcomes, critical, and important were included in the evidence profile.^10^

### Search Methods for Identification of SRs

#### Electronic Searches

We searched the “preappraised” evidence resources (defined as resources that suffered a screening process to collect only those researches with higher quality; they are regularly renewed so that the evidence we obtain through these resources is up-to-date)^16^ (from inception until September 2014); the resources are showed in the supplemental material.

We also searched the following databases from their inception until September 2014 (both MeSH and free-text terms were used): Ovid Medline, Ovid Embase, the Cochrane Database of Systematic Reviews, Psych-info, ScienceDirect, Wanfang Database, the Chinese Biomedical Literature Database (CBM), the China National Knowledge Infrastructure/China Academic Journals Full-text Database (CNKI), the TCM Database and the Chinese Scientific Journals Database (VIP). Search terms were incorporated to target stroke and SRs but intervention-specific search terms were not included since we wished to identify and include SRs of any of the TCPMs. No language restriction in the process of study search. The Medline (Ovid) search strategies are listed in Appendix 1 and were appropriately modified for both the Chinese databases using Chinese terms and other databases.

#### Other Sources of Evidence

We hand-searched the lists of references for all eligible SRs and relevant clinical guidelines. We also contacted the authors if the data were not reported sufficiently in the SR.

Grey literature resources were also researched: Stroke Engine (http://www.strokengine.ca/best_practice/acupuncture-best-practices/), Open-SIGLE (http://www.opengrey.eu/), PsycEXTRA (http://www.apa.org/psycextra/), and the National Technical Information Service (NTIS, http://www.ntis.gov/).

#### Identification of SRs

Two authors (Zhang X and Liu XT) independently screened the search results. Initially, the titles and abstracts of identified studies were reviewed so that irrelevant studies were excluded.

Then the full texts were searched and checked to confirm their eligibility. Kang DY, the third author, reviewed the full text to determine eligibility in cases of disagreement.

### Data Collection and Analysis

#### Assessment of Methodological Quality of Included SRs as Well as Identification of SRs with High Methodological Quality

Two tools, “A measurement tool to assess the methodological quality of systematic reviews” (AMSTAR)^17^ and the Oxman-Guyatt Overview Quality Assessment Questionnaire (OQAQ),^18^ were used by 2 authors (ZX and LXT) independently to rate the methodological quality of those included SRs. The quality scores were calculated in keeping with the methods and principles applied in previous researches^19–21^ for both these 2 tools as follows: 1 point was awarded when the answer was “Yes”; otherwise, 0 points were awarded. The total scores were obtained. Moreover, the reporting quality was rated by utilizing Preferred Reporting Items for Systematic Reviews and Meta-Analyses (PRISMA).^22^ To screen the SRs of high methodological quality in accordance with the “Canadian Agency for Drugs and Technologies in Health” (CADTH),^23^ we rated the SRs as “high” (range 9–11), “moderate” (range 5–8), or “low” (range 0–4) quality on the basis of overall score of AMSTAR. Studies were also assessed as being high-quality if their OQAQ overall score were ≥7. The SRs with high methodological quality were filtered into the process of data collection. Any questions occurred throughout the rating process were resolved by frequent discussions. We described the agreement validation and reliability our previous research.^24^

#### Data Extraction

Standardized data collecting forms, which were piloted on 2 SRs included in this overview, were utilized to extract data from SRs and original RCTs. One author (ZX) extracted data and a second author (LXT) examined the all extracted.

We extracted the following information from SRs: the sample sizes of each group, assessment of methodological quality, inclusion and/or exclusion criteria (study design, diagnostic criteria, the duration and state of disease, interventions studied, comparisons performed and outcomes and time points assessed) and TCM syndrome classification. The risk of bias of RCTs within included SRs was not reassessed, but instead reported keeping to the SR authors’ judgment. One researcher (ZX) rated the original RCT publication when any “Risk of bias” items were not reported in the SR and then a researcher (LXT) reassessed and checked. The following data of original RCTs were extracted: characteristics of the participants, interventions, comparisons performed and outcomes and total duration of study.

We contacted the authors of the SRs or the original study reports in the event that the required information could not be extracted from the reports. We resolved disagreements through consensus with a third author (Kang DY).

#### The GRADE Approach Step 1: Assessment of the Quality of the Evidence

The results related to the critical or important outcomes of the SRs initially recognized to being high-quality (an AMSTAR score ≥9 or an OQAQ score ≥7) evidence were used to construct the whole body of evidence. The GRADE approach^10^ was used to rate the evidence quality for each type of TCPMs, and each outcome.

For each SR, Zhang X (a clinician specialized in TCM) and Kang DY (a methodologist specialized in both clinical research methodology and systematic review) learned using the GRADE approach during the 22nd Cochrane Colloquium (Hyderabad, India, September 21th to 26th, 2014). These authors utilized the GRADE tool independently to evaluate the evidence pertaining to key outcomes. The quantitative data were analyzed to determine the whole quality of the evidence determining specific recommendations in the GRADE approach. The criteria by which evidence may be downgraded or upgraded depend on 5 methodological domains referring to risk of bias, consistency, directness, precision, and publication bias and the overall quality of the whole evidence (high, moderate, low, or very low) as well.^10^

#### The GRADE Approach step 2: From SR Evidence to Recommendations

The second component of the GRADE approach entailed the determination of the strength of the recommendations, that is, the extent to which we were confident that the positive effects of the TCPM outweighed its negative effects or vice versa.^10^ The strengths of the recommendations were classified as strong recommendations and weak recommendations (Supplemental Table I). The following 3 key factors determine the strength of the recommendations: best estimates of the effective magnitudes on both positive and negative outcomes, importance of the outcomes, and confidence in the magnitudes of estimates of the effects of the TCPM on the crucial or important outcomes.^10^

### Statistical Analysis

We defined the analyzed unit for our research is the SRs (not the individual trials). For continuous outcomes, we summarized data using the mean difference (MD) with 95% confidence interval (CI). For dichotomous outcomes, we presented the risk ratio (RR) or odds ratio (OR) and its 95% CI as appropriate. For the clinical heterogeneity among the included SRs, we did not seek to compare results across RCTs applying indirect methods such as network meta-analysis. The data were entered into EpiData 3.1^25^ and both assessment of the evidence quality and strength of the recommendations was conducted on the GRADE pro.^26^ We planned the calculation of the inter-rater reliability (Kappa coefficients)for each GRADE domain and the overall quality of evidence if sufficient SRs were included.

## RESULTS

### Identification of SRs

**FIGURE 1:**
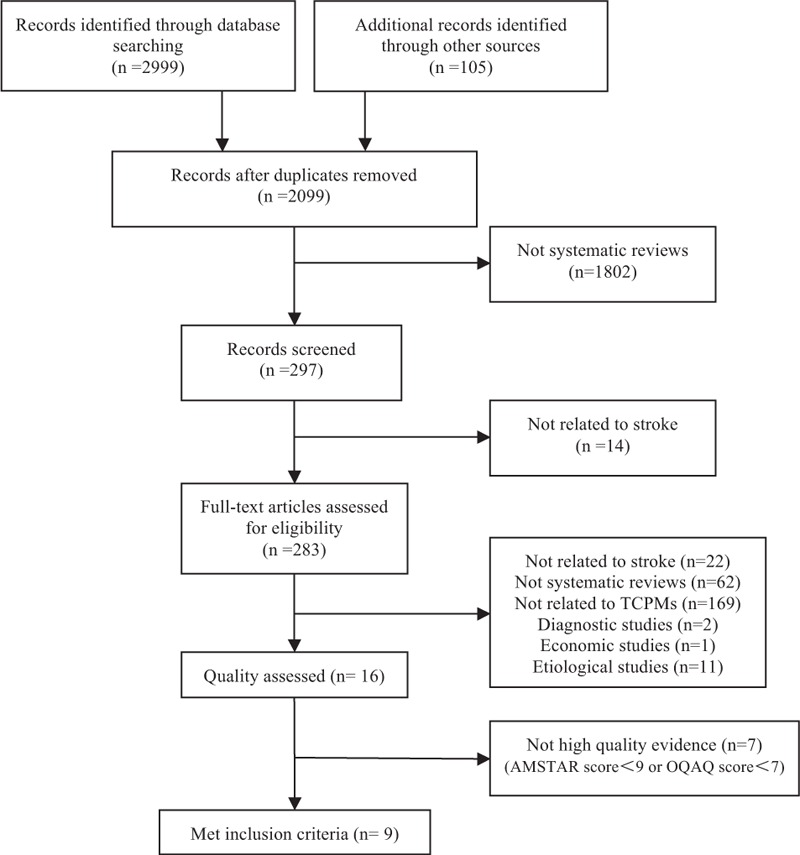
Flowchart: study selection.

The detailed literature search process and the reasons for exclusion of the studies are included in Figure 1 (PRISMA Flow Diagram).

### Description of Included Reviews

The 9 included SRs covered 83 RCTs (Supplemental table II). The following 9 TCPMs were overviewed in the SRs and varied in, frequency, duration, intensity, and comparisons: Dan Shen agents, Mailuoning, Ginkgo biloba, Dengzhanhua, Acanthopanax, Chuanxiong-type preparations, Puerarin, Huangqi, and Qing Kai Ling (Qingkailing). The characteristics including outcome measures, the effects of the interventions, the risk of bias assessment of the included RCTs and SRs are summarized in Table 1  , Supplemental table II–XII.

**TABLE 1:**
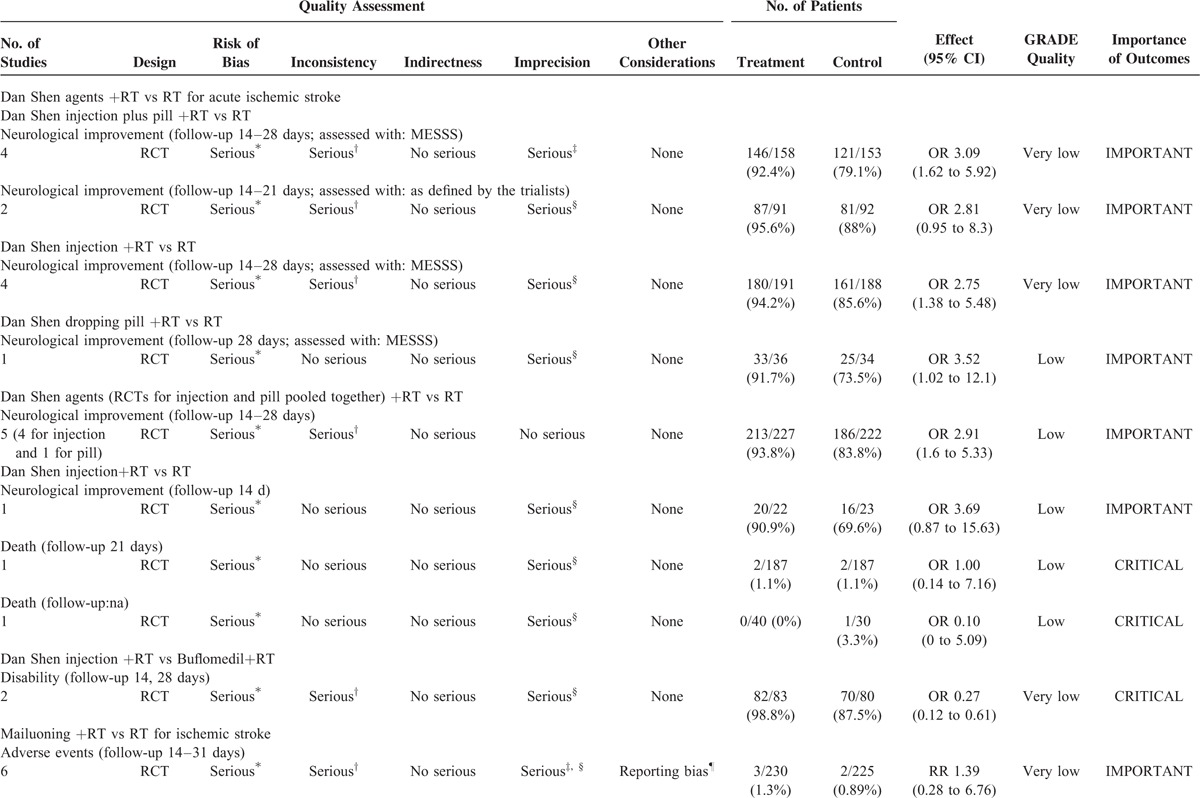
Assessment of Quality and Summarizing the Findings Using the GRADE Approach

**TABLE 1 (Continued):**
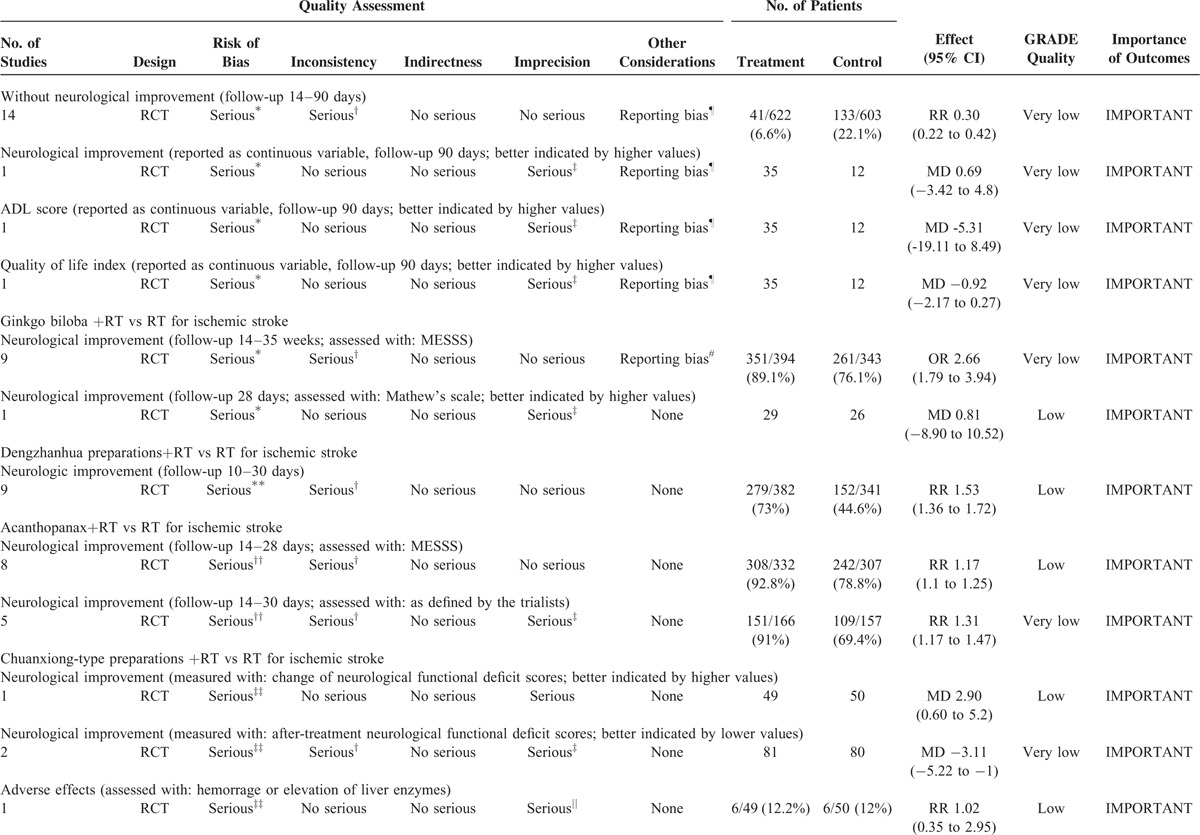
Assessment of Quality and Summarizing the Findings Using the GRADE Approach

**TABLE 1 (Continued):**
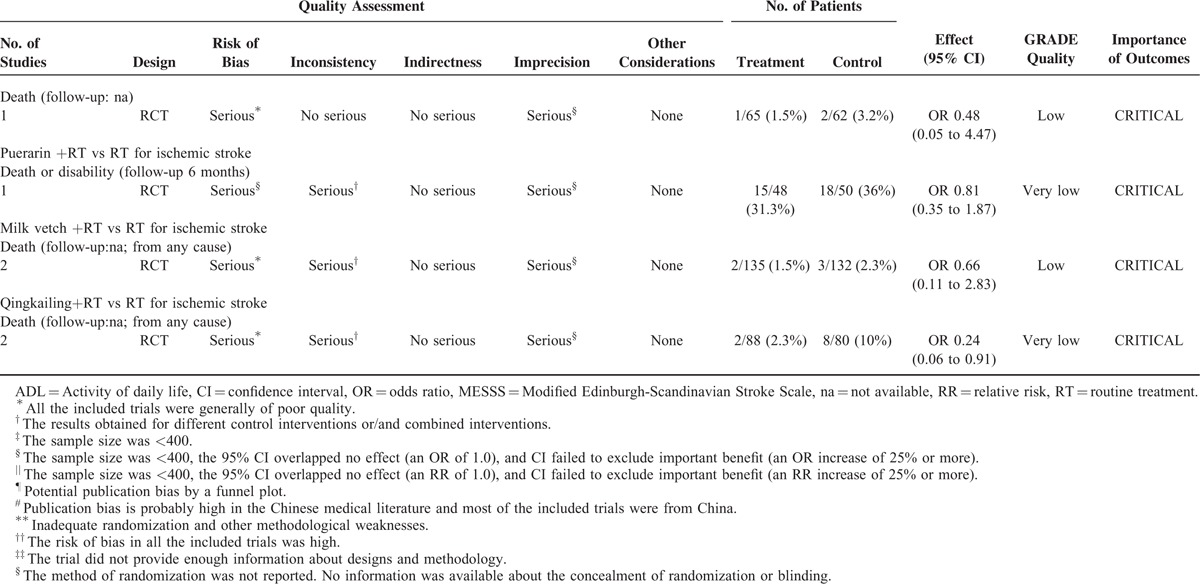
Assessment of Quality and Summarizing the Findings Using the GRADE Approach

### Quality of the Overall Body of the SR and Recommendations

#### Dan Shen Agents for Acute Ischemic Stroke

Six intervention comparisons and 9 outcomes from 3 SRs were evaluated using the GRADE approach (Table 1  ). Although the quality of the included SRs was good, all the included original RCTs were generally of poor quality due to various limitations. Therefore, the evidences for Dan Shen agents were downgraded for risk of bias by 1 level (Table 1  ). These SRs differed markedly across RCTs in duration (14 to 28 days), frequency (1 to 3 times/day) and type (injection and/or pill), and included different control interventions (e.g., snake venom, low-molecular-weight heparin, buflomedil) and combined interventions (e.g., snake venom, low-molecular-weight heparin). Therefore, 5 outcomes were considered the inconsistency to be serious. We downgraded this result by 1 quality level for imprecision (the total number of participants < 400) and the 95% CI of OR overlapped 1.0 and failed to exclude the important benefit (an OR increase of 25% or more) (Table 1  ). No serious indirectness and publication bias was observed. As a result, the quality of evidence related to the 9 critical outcomes was downgraded to either low or very low. With follow-up of 14 to 28 days, there were tiny trends toward an in neurological improvement (Modified Edinburgh-Scandinavian Stroke Scale (MESSSS): RR = 1.16, 1.10, 1.23; the outcome defined by the trialists: RR = 1.08, 1.12) (Table 2). The small relative effects (all RRs < 2)^10^ of Dan Shen and the low or very low overall quality of evidence regarding outcomes were more likely to warrant weak recommendations of Dan Shen agents as combined therapies for acute ischemic stroke (Table 2).

**TABLE 2:**
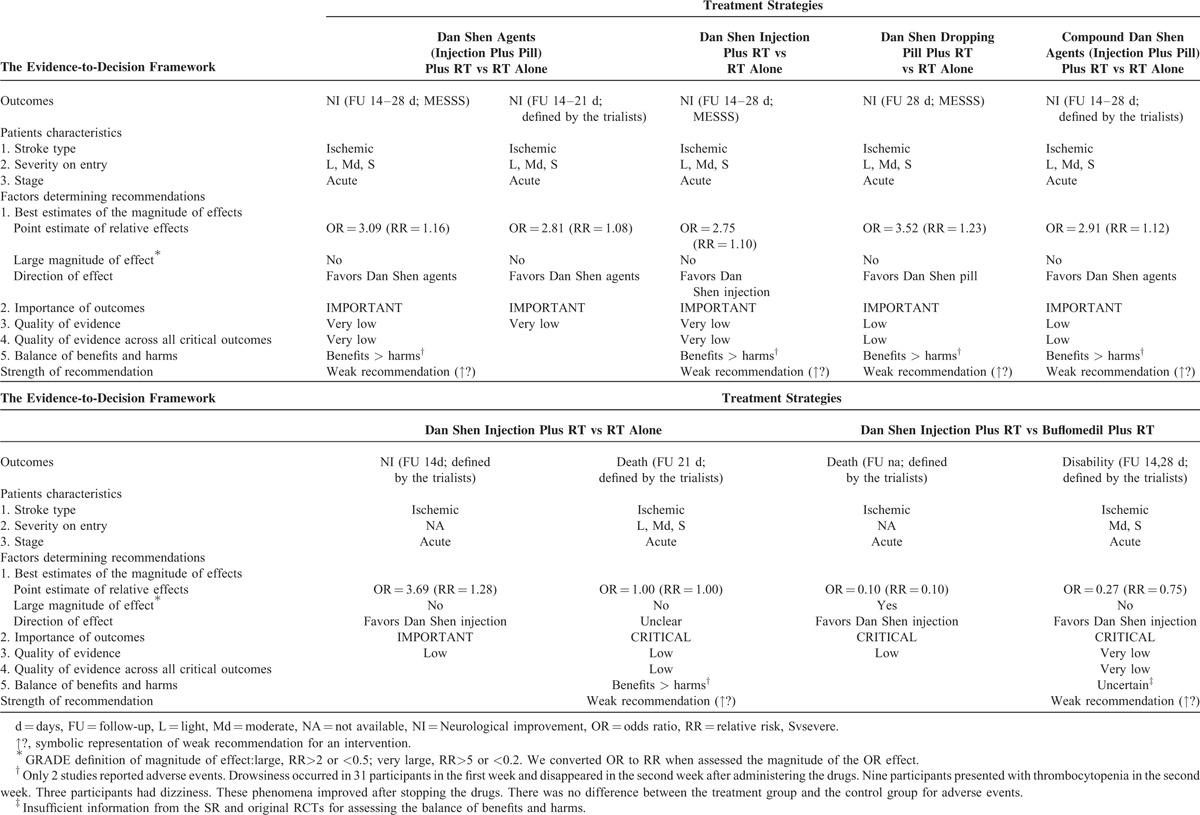
From SR Evidence to Recommendations for Dan Shen

#### Mailuoning for Acute Ischemic Stroke

One SR included 14 RCTs comparing Mailuoning plus routine therapy versus routine therapy alone. All the 5 important outcomes were considered serious study limitations because of the poor quality of included original RCTs (Table 1  ). Moreover, the trials reporting AEs and the proportion of patients without neurological improvement as outcomes used a wide variety of routine therapies (e.g., Xuesaitong injection, Kudiezi injection) and/or combined interventions (e.g., Xuesaitong injection, Xueshuanxinmaining), so significant clinical heterogeneity across the trials there was also identified (downgraded by 1 level for inconsistency). We concluded the precision of the evidence for the outcome-proportion of patients without neurological improvement was adequate, because the sample size is large (n = 1225) and the 95%CI excludes no effect (an RR of 1.0). Potential publication bias was explored by funnel plot. No serious indirectness was detected. In the end, the quality of evidence related to those 9 critical outcomes for Mailuoning was downgraded to very low. After follow-up of 14 to 90 days, a tiny trend toward an improvement in neurological (RR = 0.35, MD = 0.69) was showed (Table 3). Only 1 large relative effects^10^ (RR = 0.35), outcomes pointing in different directions (toward both favors Mailuoning and controls) and the very low overall quality of evidence were more likely to warrant weak recommendations of Mailuoning for acute ischemic stroke (Table 3).

**TABLE 3:**
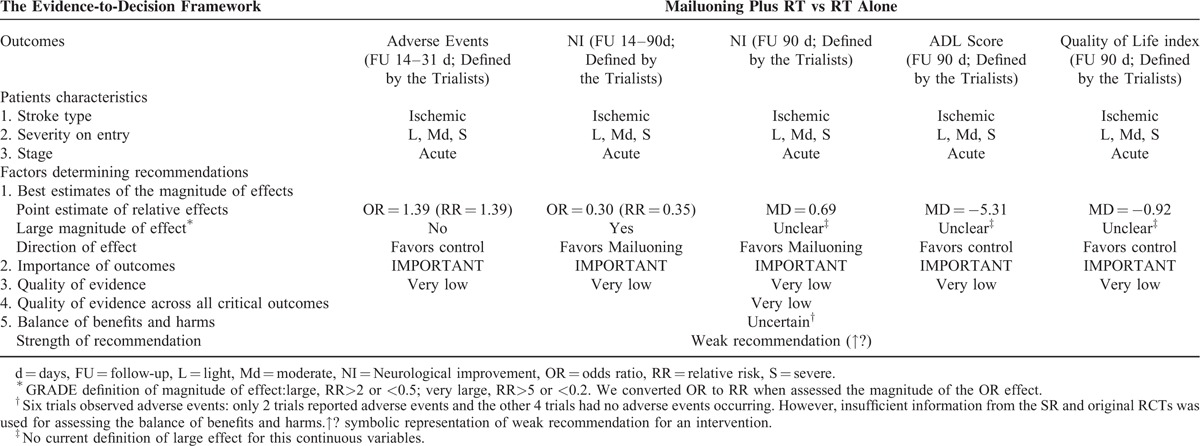
From SR Evidence to Recommendations for Mailuoning

#### Ginkgo Biloba for Acute Ischemic Stroke

One SR summarizing 2 important outcomes was assessed. For the outcome of neurological improvement (MESSS), the 9 original RCTs did not report whether they applied a blinded outcome assessment or intention-to-treat (ITT) analysis and the follow-up period was relatively short (14 to 35 days). Therefore, the quality of the evidence for this outcome was downgrade (Table 1  ). There were evidences of inconsistency of effect across RCTs for the 2 neurological improvement outcomes: considerable difference across RCTs in Ginkgo biloba preparations (Ginkgo biloba extract tablet or those for injection), control interventions (e.g., placebo, cerebralysin), and/or combined interventions (e.g., cerebralysin, venoruton, Dan Shen). Reporting bias cannot be completely ruled out because most of the included trials were from China. The original trial^27^ included in the SR reporting neurological improvement (Mathew's scale) was randomized, placebo controlled and double blind. An envelope used for keeping secret of the drug coed was utilized, which satisfied adequate concealment of randomization. However, 4 and 3 patients were lost to follow up in the Ginkgo biloba and control group respectively; these 7 patients were not excluded. Moreover, this RCT did not use ITT analysis. As a result, the quality was downgraded for this outcome. Moreover, we downgraded the quality of the evidence of this outcome for imprecision because of the small number (<400) of participants. Eventually, the evidence pertaining to these 2 outcomes was judged to be very low or low respectively (Table 4). With follow-up of 28 days to 35 weeks, there were tiny trends toward an improvement in neurological (RR = 1.18, MD = 0.81) (Table 4). The small relative effects^10^ (RR < 2) of Ginkgo biloba and the low or very low overall quality of evidence regarding outcomes were more likely to warrant weak recommendations of Ginkgo biloba for acute ischemic stroke (Table 4).

**TABLE 4:**
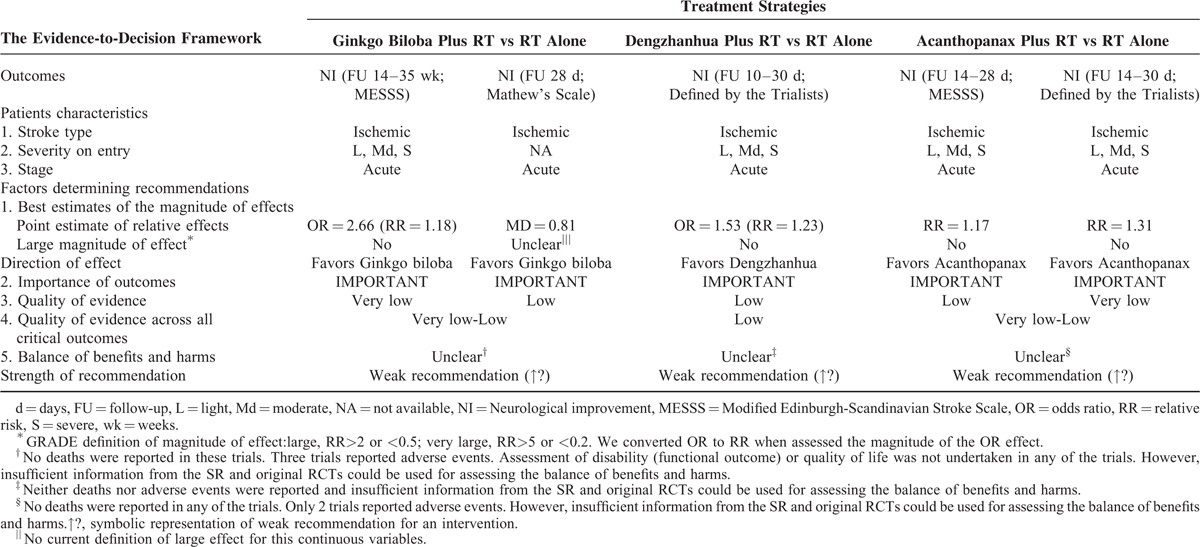
From SR Evidence to Recommendations for Ginkgo Biloba, Dengzhanhua, and Acanthopanax

#### Dengzhanhua Preparations for Acute Ischemic Stroke

One SR summarizing 1 important outcome was assessed. Inadequate randomization and other methodological weaknesses were noted among the 9 studies. Therefore, the overall quality rating was downgraded by 1 level (Table 1  ). Furthermore, difference across RCTs in duration (range 14–30 days) and dose (range 30–75 mL) without a subgroup analysis contributed to the inconsistency. As a consequence of these limitations in study design and consistency, the overall quality of evidence was judged to be low (Table 1  ). After 10 to 30 days’ follow-up, there was a tiny trend toward an improvement in neurological (RR = 1.23) (Table 4). The small relative effects^10^ (RR<2) of Dengzhanhua, the low overall evidence quality and the uncertainty in the effects of other outcomes (such as long-term harm) were more likely to warrant weak recommendations of Dengzhanhua for acute ischemic stroke (Table 4).

#### Acanthopanax for Acute Ischemic Stroke

One SR (including 13 RCTs) compared Acanthopanax plus routine therapy with routine therapy alone, of which 8 RCTs with methodological limitations reported a small positive (RR = 1.17) result on neurological improvement (MESSS) and the other 5 also reported a small positive effect (RR = 1.31) on neurological improvement (defined by the trialists). Low-quality evidence (evidence from 8 RCTs with methodological limitations, downgrade once for consistency) was demonstrated that Acanthopanax be superior to controls improving neurological function (MESSS). Additionally, very low-quality evidence (from 5 RCTs with methodological limitations, downgrade once for consistency, once for imprecision) was concluded that Acanthopanax be superior to controls in neurological improvement (defined by the trialists) (Table 1  ). The small relative effects (RR<2)^10^ of Acanthopanax, the low overall quality of evidence and the uncertainty in the effects on long-term harms were more likely to warrant weak recommendations of Acanthopanax for acute ischemic stroke (Table 4).

#### Chuanxiong-type Preparations for Acute Ischemic Stroke

One SR reported the change of neurological functional deficit scores indicating that Chuanxiong plus routine therapy was significant better than routine therapy alone (MD = 2.90). Before-after treatment neurological functional deficit scores were also evaluated indicating that Chuanxiong plus routine therapy had a superior effect than routine therapy alone (MD = -3.11). There were very-low- to low-quality evidences (evidences from the 2 RCTs with methodological limitations downgrade for inconsistency and imprecision) (Table 1  ). Furthermore, the SR suggested that no significant difference in adverse effects (RR = 1.02, 95% CI 0.35–2.95) and death (OR = 0.48, 95% CI 0.05–4.74) was found between the 2 groups. This, however, requires further investigation because the quality evidences were low (downgraded for methodological limitations, inconsistency, and imprecision). The low overall quality of evidence and the uncertain of balance between benefits and harms were more likely to warrant weak recommendations of Chuanxiong-type preparations for acute ischemic stroke (Table 5).

**TABLE 5:**
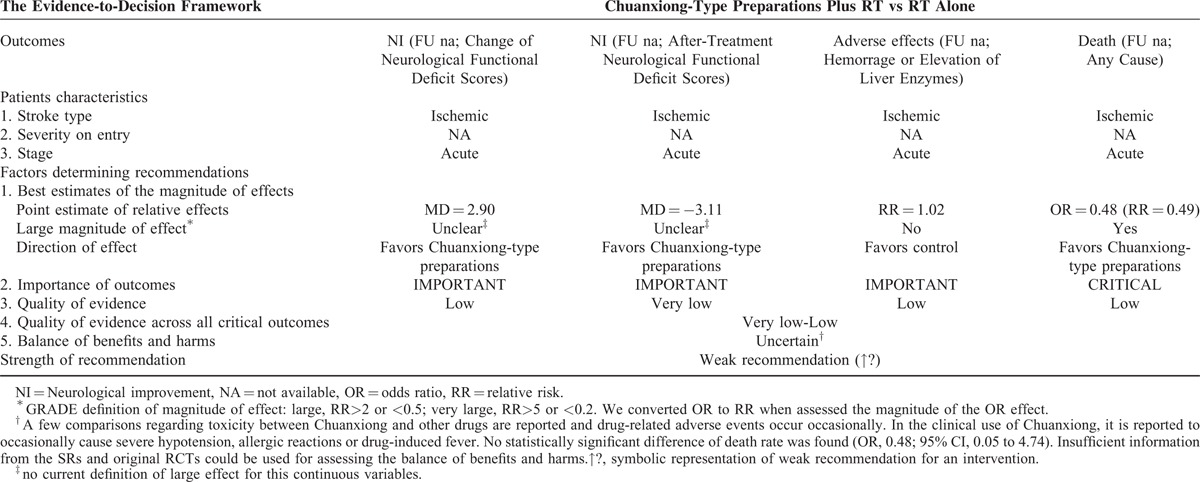
From SR Evidence to Recommendations for Chuanxiong-Type Preparations

#### Puerarin for Acute Ischemic Stroke

The rate of death or disability was evaluated and showed no significant difference indicating that Puerarin plus routine therapy did not show a better effect than routine therapy alone (OR = 0.81, 95% CI 0.35–1.87). This, however, requires further investigation because the quality evidences were low (downgraded for methodological limitations, inconsistency, and imprecision) (Table 1  ). The very low overall quality of the evidence, the small relative effect^10^ (RR<2) and the uncertain of balance between benefits and harms were more likely to warrant weak recommendations of Puerarin for acute ischemic stroke (Table 6).

**TABLE 6:**
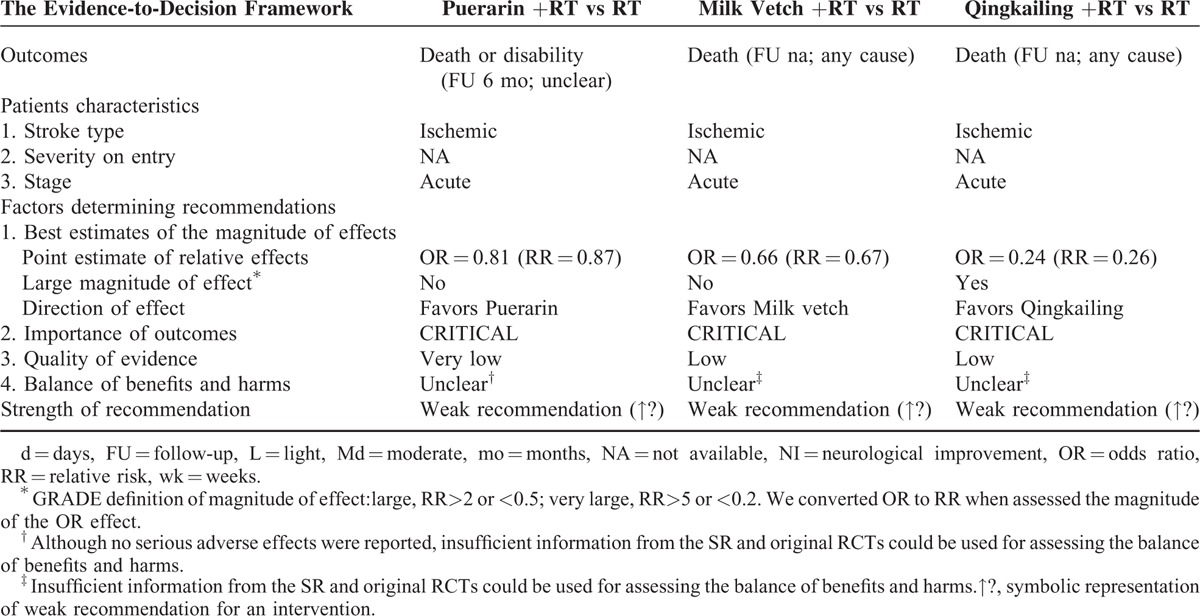
From SR Evidence to Recommendations for Puerarin, Milk Vetch and Qingkailing

#### Milk Vetch for Acute Ischemic Stroke

The rate of death was evaluated and showed no significant difference indicating that Milk vetch plus routine therapy did not had a better effect than routine therapy alone (OR = 0.66, 95% CI: 0.11–2.83). However, this requires further investigation because the quality evidences were low (downgraded for methodological limitations, inconsistency, and imprecision) (Table 1  ). The low overall quality of the evidence, the small relative effect^10^ (RR < 2) and the uncertain of balance between benefits and harms were more likely to warrant weak recommendations of Milk vetch for acute ischemic stroke (Table 6).

#### Qingkailing for Acute Ischemic Stroke

The rate of death (for any cause) was reduced in the Qingkailing group (OR = 0.24, 95% CI: 0.06–0.91). This, however, requires further investigation because the quality evidences were very low (downgraded for methodological limitations, consistency, and imprecision). The very low overall quality of the evidence and the uncertain of balance between benefits and harms were more likely to warrant weak recommendations of Qingkailing (Qing Kai Ling) for acute ischemic stroke (Table 6).

### Summary of Outcome Results Across Systematic Reviews

We summaries the results for each outcome across included Cochrane reviews in Table 7.

**TABLE 7:**
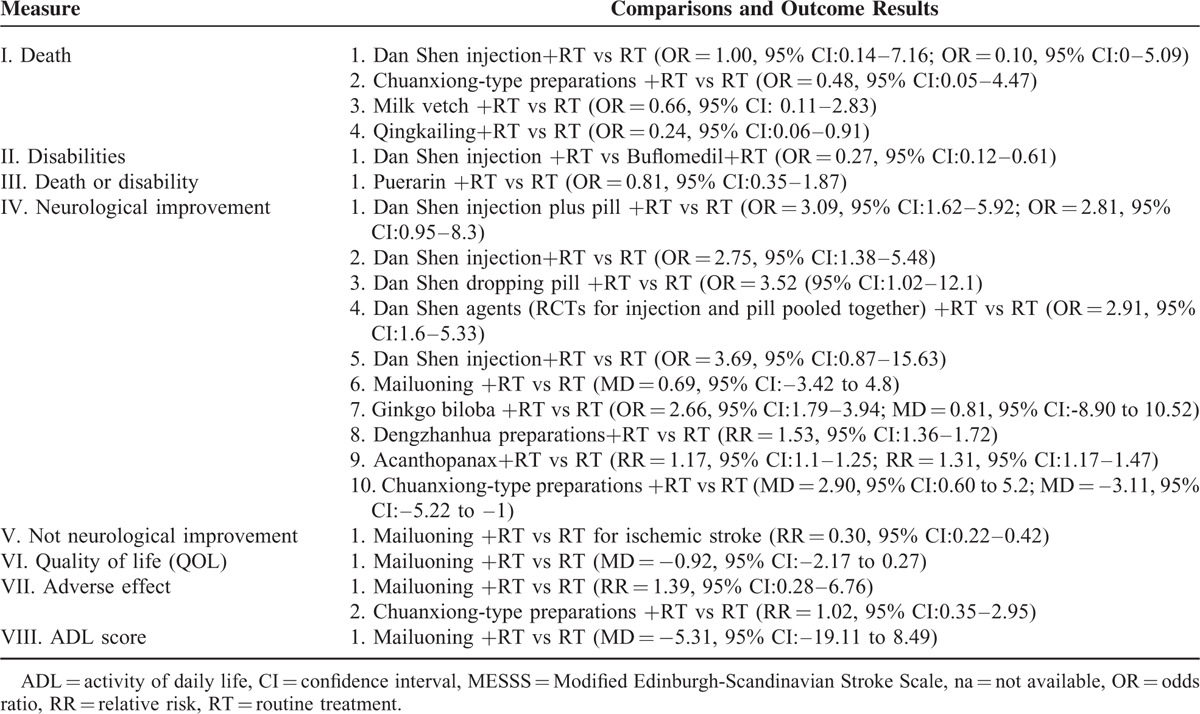
Summary of Outcome Results Across Systematic Reviews

## DISCUSSION

### Summary of Main Results

Although TCPMs has been used to treat patients suffering from stroke for many years in China, this review represents the first overview of SRs pertaining to the use of the most commonly used and Government-approved TCPMs for stroke by using the GRADE approach. This overview identified 9 SRs of RCTs that have assessed the outcomes of several aspects of the 9 TCPMs (Dan Shen agents, Mailuoning, Ginkgo biloba, Dengzhanhua, Acanthopanax, Chuanxiong-type preparations, Puerarin, Huangqi and Qing Kai Ling). In this overview, there was very limited evidence from SRs on the effect of TCPM on the important or critical outcomes. The quality of the evidence reported by the included SRs was rated using GRADE methods and ranged from very low to low. The main reasons for the quality of the evidence being downgraded include bias in the primary studies (inadequate reporting of allocation concealment and randomization methods, lack of blinding), inconsistency and imprecision. The evidence was frequently restricted to a single small trial. We finally concluded that the strength of the recommendation for all the 9 TCPMs in acute ischemic stroke was weak. These weak recommendations imply that the decision to prescribe a TCPM from the 9 TCPMs for a patient suffering from acute ischemic stroke should be approached with caution. In this study, recommendations of the domains of values and preferences or resource use were not assessed, as relevant data were unavailable.

### Overall Completeness and Applicability of Evidence

Firstly, the inclusion of both “preappraised” and non-“preappraised” evidence resources ensures that our overview serve as a comprehensive summary of all current high-quality SRs. Secondly, Our results may have been biased, as our selection process missed TCPMs with no clinical data. We can only draw conclusions regarding treatments based on available evidence. The clinical practicality of this study may be impacted by including these TCPMs and the recommendations should be revised when clinical data for these TCPMs is available. Thirdly, some included RCTs and SRs predate the last diagnostic guidelines for stroke. This increases the risk that RCTs and SRs may have included participants who would not be identified as stroke according to current diagnostic criteria and so leads a source of likely clinical heterogeneity across the evidences. Similarly, some included SRs and primary studies do not clearly report the details of their routine therapies. Critically, we identified that the consistent definition to the outcome of neurologic improvement was lack in some of the included studies. Fourthly, very little data on relatively long term (> 6 months) outcomes were identified for any TCPMs except Puerarin, which leads an important limitation of the evidence base given the chronic nature of the condition. Additionally, most of the included SRs and studies considered neurologic improvement as the primary outcome, but adverse events were often incomplete reporting. Consequently, in many assessing processes the balance between benefits and harms remains unclear. Finally, data relating to hemorrhagic stroke were not collected and analyzed; the results and conclusions of this overview should not be extended to hemorrhagic patient group. None of the studies recruited stroke patients in subacute (onset within 2–28 days) or recovery stage (onset after 28 days), so we have no effectiveness and safety information for the TCPMs in patients with stroke who onset after 2 weeks. We also have not included SRs of any TCPM as a monotherapy to treat stroke patients.

### Potential Biases in the Overview Process

While all eligible and high-quality SRs have been attempted to identify using a highly sensitive strategy, there is a possibility that some key literatures may have been overlooked, which may have impacted on our conclusions regarding some TCPMs. Moreover, an SR including low-quality RCTs may produce a misleading GRADE result, for example, yielding a narrow confidence interval (means good precision) around the incorrect intervention effect estimate. For this reason, SRs included for our study were restricted to those with high quality. Furthermore, the aim of this overview was to summary the evidence from SRs; therefore, we did not include the missing original RCTs in the SRs with lesser quality.

### Agreements and Disagreements With Other Studies or Reviews

There are no reviews comparable with this overview. However, the Evidence-Based Review of Stroke Rehabilitation (EBRSR)^28^ published clinical guidelines on the TCM treatments of people with ischemic stroke (the 16th edition, 2013), which used many of our SRs and trials. They concluded that Chinese herbal medicine may be beneficial following stroke. As the most recent search was conducted in September 2014, our overview can be considered the most up-to-date evidence on the TCPMs.

### Implications for Clinical Practice and Research

The evidence compiled by this overview weakly supports using TCPMs for acute ischemic stroke. Compared with usual care alone, the addition of Dan Shen, Mailuoning, Ginkgo biloba, Dengzhanhua, Acanthopanax and Chuanxiong-type preparations in patients with acute ischemic stroke appeared to have beneficial impact on neurological improvement, and adding Dan Shen injection or not were unclear in decreasing the rate of death and small effective in reducing disability. Mailuoning appeared to have no impact on health-related quality of life and activity of daily life. Chuanxiong-type preparations were not effective in the reduction of adverse effects, but did reduce risk of death. Puerarin, Milk vetch, Qingkailing can mildly decrease the mortality too. Well-designed and conducted SRs and RCTs are warranted to support the utilization of the 9 TCPMs on acute ischemic stroke patients.

To facilitate comparison across TCPMs SRs and the efficient future update of this overview, the following SRs of TCPMs need to standardize and unify their methods as well as reporting, such as the reporting of included RCT characteristics, risk of bias assessment criteria for both SRs and RCTs, outcomes and evidence synthesis methods.

Given that TCPM is a complex intervention and stroke is a disease treated with complex interventions, a significant challenge of SRs of TCPM is treating the potential heterogeneity among interventions (such as composition of TCPMs and methods of delivery) and outcomes (generally accepted scales or defined by the authors). Potentially effective approaches to explore this complexity include stratification ('splitting’) of outcome results or intervention types, subgroup analyses and meta-regression.

## CONCLUSIONS

The current SRs of the RCTs described the potential benefits of the 9 TCPMs (Dan Shen agents, Mailuoning, Ginkgo biloba, Dengzhanhua, Acanthopanax, Chuanxiong-type preparations, Puerarin, Huangqi, and Qing Kai Ling); however, the overall bodies of evidence were found to be of low quality. Our critical appraisal of the evidence using the GRADE approach resulted in the formulation of weak recommendation.

## Supplementary Material

Supplemental Digital Content
