# Traditional chinese patent medicine for acute ischemic stroke: an overview of systematic reviews based on the GRADE approach: Erratum

**DOI:** 10.1097/MD.0000000000007810

**Published:** 2017-08-11

In the article, “Traditional Chinese Patent Medicine for Acute Ischemic Stroke: An Overview of Systematic Reviews Based on the GRADE Approach”,^[1]^ the incorrect SDC was linked to in the article. The SDC has since been replaced with the correct file and links correctly.
