# Supplementary material for: Traditional Chinese Patent Medicine for Acute Ischemic Stroke: An Overview of Systematic Reviews Based on the GRADE Approach
Source: Medicine (Baltimore). 2016 Mar 25;95(12):e2986. doi: 10.1097/MD.0000000000002986 (PMC4998369; doi:10.1097/MD.0000000000002986)
Supplement: Supplemental Digital Content [file medi-95-e2986-s001.doc]

**Supplemental methods**

**Search strategy**

**PART ONE**: “pre-appraised” evidence resources

1. American College of Physicians Journal Club

(ACPJC, http://plus.mcmaster.ca/acpjc),

2. Evidence Update

(<http://plus.mcmaster.ca/evidenceupdates>),

3. Cochrane Library

([http://www.thecochranelibrary.com](http://www.thecochranelibrary.com/)),

4. Database of Abstracts of Reviews of Effects

(DARE, http://www.crd.york.ac.uk/crdweb/home.aspx?DB=DARE),

5. The Campbell Library (<http://www.campbellcollaboration.org/library.php>),

6. TRIP database

([http://www.tripdatabase.com](http://www.tripdatabase.com/)) and

7. Stroke

([http://stroke.ahajournals.org](http://stroke.ahajournals.org/)).

**PART TWO:**

The following search strategy,

(See: ‘Specialized register’ section in Cochrane Stroke Group: <http://onlinelibrary.wiley.com/o/cochrane/clabout/articles/STROKE/frame.html>)

using a combination of controlled vocabulary and text word terms, was used for MEDLINE and was modified to suit other databases.

MEDLINE (Ovid)

1 exp cerebrovascular disorders/

2 (stroke$ or poststroke$ or cva$).tw.

3 (cerebrovascular$ or cerebral vascular).tw.

4 (cerebral or cerebellar or brainstem or vertebrobasilar).tw.

5 (infarct$ or isch?emi$ or thrombo$ or apoplexy or emboli$).tw.

6 4 and 5

7 (cerebral or intracerebral or intracranial or parenchymal).tw.

8 (brain or intraventricular or brainstem or cerebellar).tw.

9 (infratentorial or supratentorial).tw.

10 7 or 8 or 9

11 (haemorrhage or hemorrhage or haematoma or hematoma).tw.

12 (bleeding or aneurysm).tw.

13 11 or 12

14 10 and 13

15 1 or 2 or 3 or 6 or 14

16 acupuncture/

17 exp acupuncture therapy/

18 electroacupuncture/

19 meridians/

20 acupuncture points/

21 acupuncture$.tw.

22 (electroacupuncture or electro- acupuncture).tw.

23 acupoints.tw.

24 ((meridian or non-meridian or trigger) adj10 point$).tw.

25 or/16-24

26 15 and 25

| Supplemental Table 1. The strength of recommendations | |
| --- | --- |
| Strength of recommendations | Comment |
| **Strong recommendation** |  |
| Strong recommendation for acupuncture | We are *confident* that the desirable effects of acupuncture outweigh its undesirable effects |
| Strong recommendation against acupuncture | We are *confident* that the undesirable effects of acupuncture outweigh its desirable effects |
| **Weak recommendation** |  |
| Weak recommendation for acupuncture | The desirable effects *probably* outweigh the undesirable effects |
| Weak recommendation against acupuncture | The undesirable effects *probably* outweigh the desirable effects |

| Supplemental Table II. Characteristics of included systematic reviews and their original RCTs | | | | | | | | |
| --- | --- | --- | --- | --- | --- | --- | --- | --- |
| **SRs** | **Original RCTs** | **Number of patients**(G1/G2/G3) | **Age**  (yrs) | **Gender** (M/F) | **Stroke type** | **Severity on entry** | **Time since stroke** | **Stage** |
| Wu 2006^46^ | Dai 1997^42^ | 136(46/45/45) | 48-86 | M:75% | Ischemic | L,Md,S | 3-14 mo | Recovery |
|  | Li 1997^43^ | 112(42/20/50) | 24-76 | na | Ischemic/hemorrhagic | na | 1mo-8.5yrs | Recovery |
|  | Lun 1999^44^ | 109(61/48) | 35-75 | M:60% | Ischemic/hemorrhagic | na | 2mo-5yrs | Recovery |
|  | Naeser 1992^55^ | 16(10/6) | 44-74 | na | Ischemic | Md | 1-3mo | Recovery |
|  | Wang 2001^60^ | 90(34/30/26) | 39-75 | M:56% | Ischemic/hemorrhagic | na | 2mo-5yrs | Recovery |
| Xie 2008^53^ | Han 2004^56^ | 66(34/32) | 41-79  (mean 62.1) | M:41;F:25 | ischemic/hemorrhagic | — | ＜30d | Subacute |
| Sze 2002^54^ | Hu 1993^12^ | 30(15/15) | T:Mean 63.6  (SD 6.7)  C:Mean 62.8  (SD 8) | T:15/0;  C:13/2 | ischemic/hemorrhagic | Md,S | ＜36h | Acute |
|  | Johansson 1993^57^ | 78(40/30) | Median:76 | na | Ischemic | Md,S | 4-7d | Acute |
|  | Sallstrom 1996^59^ | 45(24/21) | T:median 57  (35–69)  C:median 58  (39–72) | T:18/6;  C:16/5 | Ischemic/hemorrhagic | Md | median:40d (15-71d) | Subacute and recovery |
|  | Gosman-Hedstrom  1998^58^ | 104(37/34/33) | Weighted mean  (M 76;F:78.3) | 46/58 | Ischemic | Md,S | ＜7d | Acute |
|  | Wong 1998^60^ | 118(59/59) | 21-80 | T:38/21;  C:42/17 | Ischemic/hemorrhagic | Md,S | 10-14d | Acute |
|  | Sze 2002^61^ | 106(31/22/31/22) | mean 70.8  (SD 8.8) | 56/50 | Ischemic/hemorrhagic | Md,S | 3-15d | Acute |
|  | Tang 1996^62^ | 63(30/33) | 51-70 | 43/22 | Ischemic | na | ＜7d | Acute |
|  | Si 1998^63^ | 42(20/22) | T:mean 68  (SD 10)  C:mean 67  (SD 8) | T:15/5;  C:18/4 | Ischemic | Md | ＜7d | Acute |
|  | Jin 1999^64^ | 120(60/60) | 50-85 | 74/46 | Ischemic | na | ＜30d | Acute and subacute |
|  | Li 1999^65^ | 64(30/34) | 52-75 | T:17/13;  C:19/15 | hemorrhagic | Md | ≤2d | Acute |
|  | Zhang 1999^66^ | 241(145/96) | 35-85 | T:108/37;  C:71/25 | Ischemic/hemorrhagic | na | ＜43d | Subcute and recovery |
|  | Chou 2000^68^ | 32(16/16) | na | na | Ischemic/hemorrhagic | Md | 2d | Acute |
|  | Naeser 1992^55^ | 16(10/6) | 44-74 | na | Ischemic | Md | 1-3mo | Recovery |
|  | Johansson 2001^67^ | 150(48/51/51) | Weighted mean:76.3 | 14/136 | Ischemic | Md,S | 5-10d | Acute |
| Acute stage: stroke onset within 2 weeks of stroke onset;subacute stage:stroke onset within 2 to 28 days; recovery stage: stroke onset after 28 days. | | | | | | | | |
| Na, not available;L,light;Md,moderate;S,severe;F,female;M,male;mo,months;yrs,years;d,days;C,control group;T,treatment group;SD, standard deviation; SRs, systematic reviews;RCTs, randomized controlled trial; G1,Group1;G2,Group 2;G3,Group 3. | | | | | | | | |

| Supplemental Table II. Characteristics of included systematic reviews and their original RCTs (continued) | | | | | | |
| --- | --- | --- | --- | --- | --- | --- |
| **SRs** | **Original RCTs** | **Interventions** | | **Primary outcomes** | **Follow-up during** | **Conclusion** |
|  |  | **Treatment group** | **Control group** |  |  |  |
| Wu 2006^46^ | Dai 1997^42^ | Acupuncture+Aspirin25 mg qd;30d | Aspirin25 mg qd;30d | Neurological improvement^†^ | 30d | Positive |
|  | Li 1997^43^ | Acupuncture+PT and OT(6 times /wk,12wk) | PT and OT(6times/wk,12wk) | Neurological improvement^*^ | 3mo | Positive |
|  | Lun 1999^44^ | Acupuncture+TCM qd;45d | TCM qd;45d | Neurological improvement^†^ | 45d | Positive |
|  | Naeser 1992^55^ | Acupuncture(20 min/times,5 times/wk,1-2Hz)+ rehabilitation therapy,4wk | Sham acupuncture(20min/times,5times/wk,1-2 Hz)+ rehabilitation therapy,4wk | BMIT | 35d | Positive |
|  | Wang 2001^60^ | Acupuncture+TCM (10 times/13d,6 times) | TCM (10 times/13d,6 times) | Neurological improvement^†^ | 78d | Positive |
| Xie 2008^53^ | Han 2004^56^ | Acupuncture+basic treatments qd;63d | Basic treatments qd;63d | Swallowing function(CSRS) | — | Positive |
| Sze 2002^54^ | Hu 1993^12^ | Electrical acupuncture(30–60min/times,3times/wk,9.4 Hz),4wk | Basic treatments,4wk | SSS;  BI-100 | 7,14,21,28,90 d | SSS positive;  BI negative |
|  | Johansson 1993^57^ | Acupuncture(Classic acupuncture+Electrical acupuncture,30min/times,2times/wk, 2-5 Hz)+ PT and OT,10wk | PT and OT,10wk | Mobility Score;  BI-100 | Mobility Score:1, 3 mo;  BI:1,3,12mo | Motor negative;  BI positive |
|  | Sallstrom 1996^59^ | Acupuncture(Classic acupuncture,electrical acupuncture or moxibustion,30min/times,3-4 times/wk, 2-4Hz)+ rehabilitation therapy,6wk | Rehabilitation therapy,6wk | MAS;  SADLI | 6wk | MAS positive;  SADLI  positive |
|  | Gosman-Hedstrom  1998^58^ | Acupuncture(Classic acupuncture+electrical acupuncture,30min/times,2times/wk,2Hz)+PT and OT,10wk | PT and OT,10wk | SSS;  BI-100;  SADLI | 3wk,3mo,12mo | SSS negative;  BI negative |
|  | Wong 1998^60^ | Electrical acupuncture(30min/times,5times/wk, 20-25Hz, 10-20 mV)+PT and OT (≥2h/d),2wk | PT and OT (≥2h/d),2wk | BS;  FIM | T:mean 29.1(SD  7.9) d;  C: mean 32.4 (SD 8.2)d | BS positive; FIM positive |
|  | Sze 2002^61^ | Acupuncture [30min/times,3times/wk (outpatients) or 5 times/wk (inpatients)]+PT,OT,ST,10wk | PT,OT,ST,10wk | FMAM;  FIM;  BI-20 | 0,5,10wk | FMAM  Negative;  BI negative |
|  | Tang 1996^62^ | Acupuncture(200 times/min, acupuncture 5 mins and then 3-minute intervals,3times)+basic treatments,15d | Basic treatments,15d | Muscle power;  2nd NCDC disability score | 2,15d | 2^nd^ NCDC disability score positive |
|  | Si 1998^63^ | Acupuncture(classic acupuncture+electrical acupuncture,30min/times,5times/wk,5/45 Hz, 3.0mA)+basic treatments (heparin, low molecular dextran and nimodipine),36d | Basic treatments (heparin, low molecular dextran and nimodipine),36d | CSS | T: mean 37(SD 12)d;  C:mean 36(SD 13)d | positive |
|  | Jin 1999^64^ | Acupuncture(classic acupuncture +electrical acupuncture,50min/times,5times/wk, 5/45 Hz, 7.5mA)+basic treatments+TCM,40d | Basic treatments+TCM,40d | 2nd NCDC impairment score;  2nd NCDC disability score | 40d | 2^nd^ NCDC disability score positive |
|  | Li 1999^65^ | Acupuncture(30min/times,qd)+basic treatments | Basic treatments | 2nd NCDC impairment score;  2nd NCDC disability score | na | 2^nd^ NCDC disability score positive |
|  | Zhang 1999^66^ | Acupuncture(40min/times,qd)×20-30times+ basic treatments | Basic treatments | UNCHMS Guideline | na | UNCHMS positive |
|  | Chou 2000^68^ | Acupuncture(acupuncture 3 mins and then 5-minute inter-vals,2 times)+basic treatments,20d | Basic treatments,20d | MESSS | na | MESSS positive |
|  | Naeser 1992^55^ | Acupuncture(20 min/times,5 times/wk,1-2Hz)+ rehabilitation therapy,4wk | Sham acupuncture(20min/times,5times/wk,1-2 Hz)+ rehabilitation therapy,4wk | BMIT | 35d | BMIT negative |
|  | Johansson 2001^67^ | Acupuncture+ PT,OT,ST,10wk | G1: transcutaneous electrical nerve stimulation+PT,OT,ST,10wk,  G2: transcutaneous electrical nerve stimulation (Low intensity and high frequency:80 Hz, 0.4 mA)+PT,OT,ST,10wk | RMI;  BI-100 | 0,3,12mo | RMI negative;  BI-100 negative |
| * CSRS 1 and † CSRS 2(the two versions of Chinese Stroke Recovery Scale, based on the revised diagnostic criteria of acute cerebral infarction formulated by the second or based on principles of traditional Chinese medicine)  G1,Group1;G2,Group 2;G3,Group 3;na, not available; qd, once a day; d,day;PT, physical therapy; OT, occupational therapy; wk, week; mo, months; BMIT, Boston Motor Inventory Test; SSS, Scandinavian Stroke Scale; BI, Barthel Index; MAS, Motor Assessment Scale; SADLI, Sunnaas ADL Index; h, hours; BS, Brunnstrom Stages; FIM, Functional Independence Measure; SD, standard deviation; ST, speech therapy; FMAM, Fugl-Meyer Assessment Motor score; NCDC, National Cerebrovascular Diseases Conference; CSS, Chinese Stroke Scale; UNCHMS, Use of New Chinese Herbal Medicines in Stroke; MESSS, Modified Edinburgh-Scandinavian Stroke Scale; RMI, Rivermead Mobility Index; min, minute; TCM, traditional Chinese medicine; SRs, systematic reviews; RCTs, randomized controlled trial; CSRS, Chinese Stroke Recovery Scale. | | | | | | |

| Supplemental Table III . The strength of recommendations | |
| --- | --- |
| Strength of recommendations | Comment |
| **Strong recommendation** |  |
| Strong recommendation for acupuncture | We are *confident* that the desirable effects of acupuncture outweigh its undesirable effects |
| Strong recommendation against acupuncture | We are *confident* that the undesirable effects of acupuncture outweigh its desirable effects |
| **Weak recommendation** |  |
| Weak recommendation for acupuncture | The desirable effects *probably* outweigh the undesirable effects |
| Weak recommendation against acupuncture | The undesirable effects *probably* outweigh the desirable effects |

| Supplemental Table IV. Characteristics of included SRs and their original RCTs for Dan Shen agents | | | | | | | | |
| --- | --- | --- | --- | --- | --- | --- | --- | --- |
| **SRs** | **Original RCTs** | **Number of patients**(G1/G2) | **Age**  (yrs) | **Gender** (M/F) | **Stroke type** | **Severity on entry** | **Time since stroke** | **Stage** |
| Wu 2007^1^ | Cao 1994^2^ | 260(132/128) | T:39-81(mean 56.4);  C:41-79(mean 57.4) | T:93/39;C:91/37 | Ischemic | na | ＜14d | Acute |
|  | Mao 2001^3^ | 70(36/34) | T: mean 62(SD 7.4);  C: mean 61(SD 8.2) | T:21/15;C:22/12 | Ischemic | L,Md,S | ＜14d | Acute |
|  | Min 2004^4^ | 65(33/32) | 50-76;(mean 61.62,SD 5.68) | T:18/15;C:17/15 | Ischemic | na | ＜72h | Acute |
|  | Pan 1992^5^ | 374(187/187) | T:37-78(mean 58.28);  C:40-84(mean 58.81) | T:122/65;  C:126/61 | Ischemic | L,Md,S | ＜14d | Acute |
|  | Zhai 2001^6^ | 45(22/23) | T: mean 57(SD 9);  C: mean 59(SD 7) | T:10/12;C:14/9 | Ischemic | na | ＜14d | Acute |
|  | Zhang 2002^7^ | 80(40/40) | T: mean 61;  C: mean 62 | T:24/16;C:23/17 | Ischemic | na | ＜48h | Acute |
| Sze 2005^8^ | Bei 2001^9^ | 87(42/45) | T:37-74;  C:46-72 | T:30/12;C:32/13 | ischemic | Md,S | ＜7d | Acute |
|  | Wong 2000^10^ | 78(38/40) | T:46-72;  C:45-71 | T:21/17;C:22/18 | Ischemic | na | ＜3d | Acute |
| Wu 2007(b)^11^; | Pan 1992^5^ | 374(187/187) | T:37-78(mean 58.28);  C:40-84(mean 58.81) | T:122/65;C:126/61 | Ischemic | L,Md,S | na | Acute |
|  | Geng 2000^12^ | 70(40/30) | T: mean 59 (SD 9);  C: mean 55 (SD 5) | T:29/11;C:19/11 | Ischemic | na | mean 2.5d | Acute |
| Acute stage: stroke onset within 2 weeks of stroke onset; subacute stage: stroke onset within 2 to 28 days; recovery stage: stroke onset after 28 days. | | | | | | | | |
| Na, not available;L,light;Md,moderate;S,severe;F,female;M,male;d,days;h,hours;C,control group; T, treatment group; SD, standard deviation; SRs, systematic reviews; RCTs, randomized controlled trial. | | | | | | | | |

| Supplemental Table IV. Characteristics of included SRs and their original RCTs for Dan Shen agents (continued) | | | | | | |
| --- | --- | --- | --- | --- | --- | --- |
| **SRs** | **Original RCTs** | **Interventions** | | **Primary outcomes** | **Follow-up during** | **Conclusion** |
|  |  | **Treatment group** | **Control group** |  |  |  |
| Wu 2007^1^ | Cao 1994^2^ | Compound Dan Shen injection 20ml+Snake venom 0.5-0.75U qd×28d | Snake venom 0.5-0.75U qd×28d | Neurological Improvement^#^ | 28d | Positive |
|  | Mao 2001^3^ | Compound Dan Shen dropping pill 10×pills tid+RT×28d | RT×28d | Neurological Improvement^#^ | 28d | Positive |
|  | Min 2004^4^ | Compound Dan Shen injection 20ml+RT qd×14d | RT qd×14d | Neurological Improvement^#^ | 14d | Positive |
|  | Pan 1992^5^ | Compound Dan Shen injection 20ml+ Snake venom 0.25-0.75U qd×21d | Snake venom 0.25-0.75U qd×21d | Neurological Improvement* | 21d | Positive |
|  | Zhai 2001^6^ | Compound Dan Shen injection 30ml +RT qd×14d | RT qd×14d | Neurological Improvement* | 14d | Positive |
|  | Zhang 2002^7^ | Compound Dan Shen injection 20ml qd×14d+ low-molecular-weight heparin 5000U bid×7d | low-molecular-weight heparin 5000U bid×7d | Neurological improvement^#^ | 14d | Positive |
| Sze 2005^8^ | Bei 2001^9^ | Buflomedil 300mg+10% glucose iv×14d | Compound Dan Shen injection 20ml+low molecular weight Dextrane iv×14d | Disability | 14d | Positive |
|  | Wong 2000^10^ | Buflomedil 150mg+0.9% NaCl iv×14d | Compound Dan Shen injection 30ml+0.9%NaCl iv×14d | Disability | 28d | Positive |
| Wu 2007(b)^11^ | Pan 1992^5^ | Compound Dan Shen injection 20ml+Snake venom 0.25-0.75U qd×21d | Snake venom 0.25-0.75U qd×21d | Neurological Improvement*;  Mortality | 21d | Positive |
|  | Geng 2000^12^ | Compound Dan Shen injection 20m+5% glucose 300ml iv+RT×6-9w | Low-molecular-weight heparin 500ml iv+ RT×6-9w | Mortality | na | Positive |
| na, not available; qd, once a day; bid, twice a day; d, days; w, weeks;RT,routine treatment; SRs, systematic reviews; RCTs, randomized controlled trial; FDP, fructose diphosphate ;PGE, prostaglandin E;iv, intravenous drip.  *As defined by the trialists.  ^#^ MESSS score. | | | | | | |

| Supplemental Table V. Characteristics of included SRs and their original RCTs for Mailuoning | | | | | | | | | | | | | |
| --- | --- | --- | --- | --- | --- | --- | --- | --- | --- | --- | --- | --- | --- |
| **SRs** | **Original RCTs** | | **Number of patients**(G1/G2) | | **Age**  (yrs) | **Gender** (M/F) | | **Stroke type** | **Severity on entry** | | **Time since stroke** | | **Stage** |
| Yang 2009^13^ | Ding 2005^14^ | | 106 (53/53) | | T:42-82;  C:43-81 | na | | Ischemic | na | | ＜3d | | Acute |
|  | Guo 2002^15^ | | 148 (76/72) | | T:44-87;  C:46-85 | T:46/30;  C:44/28 | | Ischemic | L,Md,S | | ＜3d | | Acute |
|  | He 2001^16^ | | 67 (34/33) | | T:48-78;  C:51-79 | T:23/11;  C 20/13 | | Ischemic | L,Md,S | | ＜48h | | Acute |
|  | Huang 2006^17^ | | 70 (38/32) | | T: mean 65.0(SD 6.8);  C: mean 64.0(SD 8.4) | T:22/16;  C:18/14 | | Ischemic | L,Md,S | | ＜7d | | Acute |
|  | Mu 1998^18^ | | 64(32/32) | | T: mean 46(SD 8.1);  C: mean 49(SD 8.2) | T:23/9;  C:20/12 | | Ischemic | L,Md,S | | ＜7d | | Acute |
|  | Sun 2002^19^ | | 100(50/50) | | T: mean 42(SD 8.2);  C: mean 43(SD 8.1) | T:31/19;  C: na | | Ischemic | na | | ＜3d | | Acute |
|  | Wang 2007^20^ | | 60(32/38) | | T:51-80;  C:51-80 | T:19/13;  C:16/12 | | ischemic | L,Md,S | | ＜3d | | Acute |
|  | Xu 1999^21^ | | 70(36/34) | | T:50-72;  C:49-72 | T:25/11;  C:24/10 | | Ischemic | na | | ＜3d | | Acute |
|  | Yan 2001^22^ | | 72(36/36) | | T:43-80;  C:42-81 | T:22/14;  C:24/12 | | Ischemic | L,Md,S | | ＜7d | | Acute |
|  | Yan 2003^23^ | | 90(45/45) | | T:46-70;  C:52-71 | T:32/13;  C:31/14 | | Ischemic | na | | ＜24h | | Acute |
|  | Yang 2007^24^ | | 104(52/52) | | T:45-72;  C:43-75 | T:30/22;  C:28/24 | | Ischemic | L,Md,S | | ＜3d | | Acute |
|  | Yu 2006* | | 55(40/15) | | T:mean 68.48(SD 8.96);  C:mean 66.67(SD 7.10) | T:20/20;  C:9/6 | | Ischemic | L,Md,S | | ＜3d | | Acute |
|  | Zhang 1997^25^ | | 132(66/66) | | T:41-72;  C:41-72 | T:44/22;  C:46/20 | | Ischemic | L,Md,S | | ＜4d | | Acute |
|  | Zhang 2004^26^ | | 80(41/39) | | T: mean 62.29(SD 6.12);  C: mean 61.98(SD 6.02) | T:24/17;  C:22/17 | | Ischemic | na | | ＜3d | | Acute |
|  | Zhou 2004^27^ | | 62(31/31) | | T:45-76;  C:45-76 | na | | Ischemic | na | | ＜48h | | Acute |
| Acute stage: stroke onset within 2 weeks of stroke onset; subacute stage: stroke onset within 2 to 28 days; recovery stage: stroke onset after 28 days. | | | | | | | | | | | | | |
| Na, not available;L,light;Md,moderate;S,severe;F,female;M,male;d,days;h,hours;C,control group;T,treatment group;SD, standard deviation; SRs, systematic reviews; RCTs, randomized controlled trial;G1,group 1;G2,group 2.  *not available reference because of the wrong quotation in the SR. | | | | | | | | | | | | | |
| Supplemental Table V. Characteristics of included SRs and their original RCTs for Mailuoning (continued) | | | | | | | | | | | | | |
| **SRs** | | **Original RCTs** | | **Interventions** | | | | | | **Primary outcomes** | | **Follow-up during** | **Conclusion** |
|  | |  | | **Treatment group** | | | **Control group** | | |  | |  |  |
| Yang 2009^13^ | | Ding 2005^14^ | | Mailuoning 20ml iv×28d+RT | | | RT×28d | | | Neurological improvement^#^ | | 31d | Positive |
|  |  | Guo 2002^15^ | | Mailuoning 20ml iv+Nimodipine 8mg iv×14d | | | Nimodipine 8mg iv×14d | | | Neurological improvement^#^ | | 14d | Positive |
|  |  | He 2001^16^ | | Mailuoning 20ml qd iv+Enoxaparin 0.4ml bid subcutaneously×14d | | | Enoxaparin 0.4 ml bid subcutaneously×14d | | | Neurological improvement^#^; FAE | | 14d | Positive |
|  |  | Huang 2006^17^ | | Mailuoning 20ml iv qd+low molecular weight heparin 5000U qd subcutaneously×14d | | | Low molecular weight heparin 5000U qd subcutaneously×14d | | | Neurological improvement^#^ | | 14d | Positive |
|  |  | Mu 1998^18^ | | Mailuoning 20ml iv qd+ FDP 10g iv qd×20d | | | FDP 10g iv qd×20d | | | Neurological improvement^#^ | | 20d | Positive |
|  |  | Sun 2002^19^ | | Mailuoning 20ml iv+RT×28d | | | RT×20d | | | Neurological improvement^#^ | | 31d | Positive |
|  | | Wang 2007^20^ | | Mailuoning 20ml iv qd+RT×14d | | | RT×14d | | | Neurological improvement^#^;FAE | | 14d | Positive |
|  |  | Xu 1999^21^ | | Mailuoning 20ml iv qd+RT×28d | | | RT×28d | | | Neurological improvement^#^ | | 28d | Positive |
|  |  | Yan 2001^22^ | | Mailuoning 20ml iv qd+RT×14d | | | RT×14d | | | Neurological improvement^#^;FAE | | 28d | Positive |
|  |  | Yan 2003^23^ | | Mailuoning 20ml iv+Xuesaitong injection 400mg×14d | | | Xuesaitong injection 400mg×14d | | | Neurological improvement^#^;FAE | | 14d | Positive |
|  |  | Yang 2007^24^ | | Mailuoning 20ml iv qd+ kudiezi injection 400mg×14d 40ml×28d | | | Kudiezi injection 40ml×28d | | | Neurological improvement^#^;FAE | | 31d | Positive |
|  |  | Yu 2006* | | Mailuoning 20ml qd+RT×14d | | | Placebo+RT×14d | | | MESSS score;  ADL score; QLI | | 3mo | Positive |
|  |  | Zhang 1997^25^ | | Mailuoning 20ml iv qd+Xueshuanxinmaining 4 pills tid×18-30d | | | Xueshuanxinmaining 4 pills tid×18-30d | | | Neurological improvement^#^ | | 38d | Positive |
|  |  | Zhang 2004^26^ | | Mailuoning 20ml iv qd+Buyanghuanwutang×30d | | | Buyanghuanwutang×30d | | | Neurological improvement^#^ | | 35d | Positive |
|  |  | Zhou 2004^27^ | | Mailuoning 20ml iv qd+Flunarizine 5mg bid×20d | | | Flunarizine 5mg bid×20d | | | Neurological improvement^#^;FAE | | 30d | Positive |
| qd, once a day; bid,twice a day;d,days;mo,months;RT,routine treatment;SRs, systematic reviews; RCTs, randomized controlled trial; FDP,fructose diphosphate; iv, intravenous drip; FDP: Fructose-1,6-Diphosphate;FAE, Frequency of adverse events; MESSS, Modified Edinburgh-Scandinavian Stroke Scale; ADL, activity of daily life; QLI: quality of life index.  *not available reference because of the wrong quotation in the SR.  ^#^number of patients with neurological improvement | | | | | | | | | | | | | |

| Supplemental Table VI. Characteristics of included SRs and their original RCTs for Ginkgo biloba | | | | | | | | |
| --- | --- | --- | --- | --- | --- | --- | --- | --- |
| **SRs** | **Original RCTs** | **Number of patients**(G1/G2) | **Age**  (yrs) | **Gender** (M/F) | **Stroke type** | **Severity on entry** | **Time since stroke** | **Stage** |
| Zeng 2005^28^ | Feng 2002^29^ | 106 (53/53) | 67(35/32) | T: mean 63.1;  C: mean 60.6 | Ischemic | na | 6-48h | Acute |
|  | Garg 1995^30^ | 148 (76/72) | 55(29/26) | ― | Ischemic | na | ≥48h and <2w | Acute |
|  | Hu 1999^31^ | 67 (34/33) | 114(64/50) | T:44-85;  C42-86 | Ischemic | na | 1d-3wk,mean 10d | Acute |
|  | Li 1998^32^ | 70 (38/32) | 87(44/43) | T:41-73;  C:40-75 | Ischemic | L,Md,S | ＜8d | Acute |
|  | Li 2001^33^ | 64(32/32) | 61(32/29) | 49-80 | Ischemic | L,Md,S | <2w | Acute |
|  | Li 2003^34^ | 100(50/50) | 93(47/46) | T: mean 61.2(SD 19.3);  C: mean 56.4(SD 18.1) | Ischemic | na | <2w | Acute |
|  | Song 2001^35^ | 60(32/38) | 84(42/42) | T:36-82;  C:35-79 | ischemic | na | <2w | Acute |
|  | Tu 2000^36^ | 70(36/34) | 60(40/20) | T:47-81;  C:44-78 | Ischemic | na | 24-72h | Acute |
|  | Yuan 2002^37^ | 72(36/36) | 68(38/30) | T: mean 59.3;  C: mean 57.4 | Ischemic | na | <2w | Acute |
|  | Zhu 2001^38^ | 90(45/45) | 32(31/31) | T: mean 65;  C: mean 67 | Ischemic | na | ＜48h | Acute |
| Acute stage: stroke onset within 2 weeks of stroke onset; subacute stage: stroke onset within 2 to 28 days; recovery stage: stroke onset after 28 days. | | | | | | | | |
| Na, not available;L,light;Md,moderate;S,severe;F,female;M,male;d,days;h,hours;w,weeks;C,control group; T, treatment group; SD, standard deviation; SRs, systematic reviews; RCTs, randomized controlled trial;G1,group 1;G2,group 2. | | | | | | | | |

| Supplemental Table VI. Characteristics of included SRs and their original RCTs for Ginkgo biloba (continued) | | | | | | |
| --- | --- | --- | --- | --- | --- | --- |
| **SRs** | **Original RCTs** | **Interventions** | | **Primary outcomes** | **Follow-up during** | **Conclusion** |
|  |  | **Treatment group** | **Control group** |  |  |  |
| Zeng 2005^28^ | Feng 2002^29^ | GBET 40mg tid+RT(hydroxyethyl starch + venoruton + diphosphate choline)×28d | RT×28d | Neurological improvement^#^ | 35d | Positive |
|  | Garg 1995^30^ | GBET 40mg qid×28d | Placebo×28d | Neurological deficit* | 28d | Negative |
|  | Hu 1999^31^ | GBET 80mg tid+Cerebralysin×20d | Cerebralysin×20d | Neurological improvement^#^ | 20d | Positive |
|  | Li 1998^32^ | GBET 80mg tid+RT(dextran 40 + venoruton + CXQ) | RT | Neurological improvement^#^ | 21d | Positive |
|  | Li 2001^33^ | GBET 40 mg tid+RT (citicoline + dextran 40 + danshen)×20d | RT×20d | Neurological improvement^#^ | 20d | Positive |
|  | Li 2003^34^ | GBEI 52.5mg(15 ml) iv qd+RT(venoruton + aspirin)×15d | RT×15d | Neurological improvement^#^ | 28d | Positive |
|  | Song 2001^35^ | GBEI 70.0mg(20 ml) iv+RT(CDSI + venoruton + dextran 40)×20-30d | RT×20-30d | Neurological improvement^#^ | 30d | Positive |
|  | Tu 2000^36^ | GBET 40mg tid+RT×28d | RT×28d | Neurological improvement^#^ | 28d | Positive |
|  | Yuan 2002^37^ | GBEI 70.0mg(20 ml) iv + RT(aspirin 100 mg + diphosphate choline)×20d | RT×20d | Neurological improvement^#^ | 20d | Positive |
|  | Zhu 2001^38^ | GBEI 70.0 mg (20ml) iv qd+ RT aspirin 100 mg qd and dextran 40 500 ml iv qd)×14d | RT×14d | Neurological improvement^#^ | 14d | Positive |
| na, not available; qd, once a day; tid, three times a day;qid,four times a day;d,days;w, weeks;mo, months; iv, intravenous drip; SRs, systematic reviews; RCTs, randomized controlled trial;GBET,Ginkgo biloba extract tablet;GBEI,Ginkgo biloba extract injection; RT,routine treatment;CXQ,chuan xiong qin (herb); CDSI, compound dan shen injection (herbs).  ^#^ MESSS;*Mathew’scale. | | | | | | |

| Supplemental Table VII. Characteristics of included SRs and their original RCTs for Dengzhanhua preparations | | | | | | | | |
| --- | --- | --- | --- | --- | --- | --- | --- | --- |
| **SRs** | **Original RCTs** | **Number of patients**(G1/G2) | **Age**  (yrs) | **Gender** (M/F) | **Stroke type** | **Severity on entry** | **Time since stroke** | **Stage** |
| Cao 2008^39^ | Feng 2005^40^ | 108(58/50) | T:45-78(mean 62.8);  C:49-76(mean 63.5) | T:39/19;  C:33/17 | Ischemic | na | ＜3d | Acute |
|  | Huang 2005^41^ | 60(30/30) | T:50-82(mean 63,SD 6.78);  C:53-91(mean 65,SD 6.39) | T:20/10;  C:19/11 | Ischemic | L,Md,S | 1-7d | Acute |
|  | Li 1996^42^ | 80(40/40) | T: mean 64.5(SD 7.8);  C: mean 62.9(SD 9.1) | T:19/22;  C:15/24 | Ischemic | na | ＜3d | Acute |
|  | Liu 2004a^43^ | 106(56/50) | T:52-80(mean 64; SD 6);  C:49-81(mean 63,SD 5) | T:30/26;  C:26/24 | Ischemic | na | ＜7d | Acute |
|  | Peng 2005^44^ | 66(33/33) | T:52-76(mean 64)  C:50-74(mean 62) | T:20/13;  C:21/12 | Ischemic | na | T:8-110h (mean 59);  C:9-106h (mean 57.5) | Acute |
|  | Wang 2004^45^ | 120(64/56) | T: mean 62.4;  C: mean 64.6 | T:40/24;  C:40/16 | Ischemic | L,Md,S | T: mean 40.41 (SD 20.23)h;  C: mean 40.38 (SD 28.35)h | Acute |
|  | Wang 2005^46^ | 68(35/33) | T:53-89(mean 69.7,SD 5.4)  C:55-87(mean 67.8,SD 5.2) | T:22/13;  C:21/12 | Ischemic | na | T:8-71h (mean 22.6;SD 9.8);  C:9-68h (mean 21.7;SD 8.9); | Acute |
|  | Wen 2003^47^ | 69(39/30) | T: mean 66.2;  C: mean 65.7 | T:26/13  C:20/10 | Ischemic | L,Md,S | T: mean 45.41h (SD 26.23);  C: mean 45.38h (SD 36.35) | Acute |
|  | Yu 2005^48^ | 46(26/20) | na | na | Ischemic | na | ＜24h | Acute |
| Acute stage: stroke onset within 2 weeks of stroke onset;subacute stage:stroke onset within 2 to 28 days; recovery stage: stroke onset after 28 days. | | | | | | | | |
| Na, not available;L,light;Md,moderate;S,severe;F,female;M,male;d,days;h,hours; C,control group; T, treatment group; SD, standard deviation; SRs, systematic reviews; RCTs, randomized controlled trial;G1,group 1;G2,group 2. | | | | | | | | |

| Supplemental Table VII. Characteristics of included SRs and their original RCTs for Dengzhanhua preparations (continued) | | | | | | |
| --- | --- | --- | --- | --- | --- | --- |
| **SRs** | **Original RCTs** | **Interventions** | | **Primary outcomes** | **Follow-up during** | **Conclusion** |
|  |  | **Treatment group** | **Control group** |  |  |  |
| Cao 2008^39^ | Feng 2005^40^ | Dengzhanhua 40ml+RT qd×14d | RT qd×14d | Neurologic Improvement | 30d | Positive |
|  | Huang 2005^41^ | Dengzhanhua 40ml+RT qd×14d | RT qd×14d | Neurologic Improvement | 14d | Positive |
|  | Li 1996^42^ | Dengzhanhua 16ml+RT qd×10d | RT qd×10d | Neurologic Improvement | 10d | Positive |
|  | Liu 2004a^43^ | Dengzhanhua 75mg+RT qd×14d | RT qd×14d | Neurologic Improvement | 14d | Positive |
|  | Peng 2005^44^ | Dengzhanhua 40ml+RT qd×14d | RT qd×14d | Neurologic Improvement | 14d | Positive |
|  | Wang 2004^45^ | Dengzhanhua 30mg+RT qd×30d | RT qd×30d | Neurologic Improvement | 30d | Positive |
|  | Wang 2005^46^ | Dengzhanhua 40ml+RT qd×15d | RT qd×15d | Neurologic Improvement | 15d | Positive |
|  | Wen 2003^47^ | Dengzhanhua 30ml+RT qd×28d | RT qd×28d | Neurologic Improvement | 28d | Positive |
|  | Yu 2005^48^ | Dengzhanhua 40ml+RT qd×30d | RT qd×30d | Neurologic Improvement | 30d | Positive |
| qd, once a day; d, days; w, weeks; RT, routine treatment; SRs, systematic reviews; RCTs, randomized controlled trial. | | | | | | |

| Supplemental Table VIII. Characteristics of included SRs and their original RCTs for Acanthopanax | | | | | | | | | | | |
| --- | --- | --- | --- | --- | --- | --- | --- | --- | --- | --- | --- |
| **SRs** | **Original RCTs** | Number of patients(G1/G2) | **Stroke type** | **Severity on entry** | **Time since stroke** | **Stage** | **Interventions** | | **Primary outcomes** | **Follow-up during** | **Conclusion** |
|  |  |  |  |  |  |  | **Treatment group** | **Control group** |  |  |  |
| Li 2009^49^ | He 1998^50^ | 70(36/34) | Ischemic | L,M,S | na | Acute | Acanthopanax 40ml qd + Nimodipine 20 mg tid+RT×14d | Nimodipine 20mg tid+RT×14d | Neurological improvement# | 14d | Positive |
|  | Jia 1998^51^ | 62(31/31) | Ischemic | na | 3h-1wk | Acute | Acanthopanax 50ml + Hydroxyethy starch 500ml+ Danshen 20ml qd×28d | Hydroxyethy starch 500ml+ Danshen 20ml qd×28d | Neurological Improvement* | 28d | Positive |
|  | Li 2007^52^ | 93(52/41) | Ischemic | na | ＜72h | Acute | Acanthopanax 30ml qd×10db + Defibrase (10 u at first dosage  and afterwards 5 u qod, 3 times in all)+RT | Defibrase (10 u at first dosage  and afterwards 5 u qod, 3 times in all)+RT | Neurological improvement^#^ | 28d | Positive |
|  | Long 2000^53^ | 60(30/30) | Ischemic | na | 6h-3d | Acute | Acanthopanax 80ml qd+ urokinase 0.1 million units bid×14d | RT bid×14d | Neurological Improvement* | 14d | Positive |
|  | Peng 2006^54^ | 100(50/50) | Ischemic | na | 6h-3d | Acute | Acanthopanax 250ml+ RT qd ×14d | RT qd ×14d | Neurological improvement^#^ | 14d | Positive |
|  | Qin 2001^55^ | 86(43/43) | Ischemic | na | ＜24h | Acute | Acanthopanax 60ml qd + Defibrase (10 u at first dosage and afterwards 5 u qod, 4 times in all)+RT×14d | Defibrase (10 u at first dosage  and afterwards 5 u qod, 4 times in all)+Routine treat-ment×14d | Neurological improvement# | 14d | Positive |
|  | Wang 2006^56^ | 60(30/30) | Ischemic | na | na | Acute | Acanthopanax 80ml+ RT qd ×15d | RT qd×15d | Neurological improvement# | 15d | Positive |
|  | Wen 2005^57^ | 83(43/40) | Ischemic | na | ＜1wk | Acute | Acanthopanax 60ml+2.5ATA RT 80min(with having  a rest for 20 minutes every 40 minute) qd+RT×15d | 2.5ATA RT 80min(with having  a rest for 20 minutes every 40 minute) qd+Routine treat-ment×15d | Neurological improvement* | 15d | Positive |
|  | Wu 2000^58^ | 78(42/36) | Ischemic | L,M,S | na | Acute | Acanthopanax 60ml + Buflomedil 200mg qd×20d | Buflomedil 200mg qd×20d | Neurological improvement* | 20d | Positive |
|  | Xiao 2007^59^ | 62(37/25) | Ischemic | na | na | Acute | Acanthopanax 250ml+ Buflomedil 150 mg qd+RT×28d | Buflomedil 150mg qd+RT×28d | Neurological improvement^#^ | 28d | Positive |
|  | Xu 2003^60^ | 112(56/56) | Ischemic | L,M,S | ＜48h | Acute | Acanthopanax 60ml qd+ Low-molecular heparin 0.4ml bid+RT×14d | Low-molecular heparin 0.4ml bid+RT×14d | Neurological improvement^#^ | 14d | Positive |
|  | Ye 2003^61^ | 40(20/20) | Ischemic | na | ＜2wk | Acute | Acanthopanax 60-80ml + RT qd×30d; | RT qd×30d | Neurological improvement* | 30d | Positive |
|  | Yu 2002^62^ | 56(28/28) | Ischemic | na | na | Acute | Acanthopanax 40-60ml + RT qd×14d | RT qd×14d | Neurological improvement^#^ | 14d | Positive |
| Acute stage: stroke onset within 2 weeks of stroke onset;subacute stage:stroke onset within 2 to 28 days; recovery stage: stroke onset after 28 days.  na, not available;L,light;Md,moderate;S,severe;F,female;M,male;d,days;h,hours; qd,once a day; qod, once every 2 days; bid,twice a day;wk,weeks;C,control group;T,treatment group; RT, routine treatment;SD, standard deviation; SRs, systematic reviews; RCTs, randomized controlled trial;G1,group 1;G2,group 2;ATA, Atmosphere Absolute.  *Defined by the trialists’ own definition, which is similar to MESSS. ^#^ MESSS score. | | | | | | | | | | | |

| Supplemental Table IX. Characteristics of included SRs and their original RCTs for Chuanxiong-type preparations | | | | | | | | | |
| --- | --- | --- | --- | --- | --- | --- | --- | --- | --- |
| **SRs** | **Original RCTs** | **Number of patients(G1/G2)** | **Age**  **(yrs)** | **Gender (M/F)** | **Stroke type** | | **Time since stroke** | | **Stage** |
| Yuan 2008^63^ | Fan 2003^64^ | 62(32/30) | T:50-78(mean 61.2,SD 13.4);  C:52-76(mean 60.3,SD 14.7) | T:24/8;  C:21/9 | Ischemic | | ＜5d | | Acute |
|  | Wang 2006^65^ | 99(49/50) | 53-87(mean 66.7,SD 8.3) | 53/46 | Ischemic | | 3-72h | | Acute |
| Wu 2007(b)^66^ | Guo 2002^15^ | 127(65/62) | T:41-80(mean 62.8);  C:39-79(mean 63.1) | T:36/29  C:34/28 | Ischemic | | ＜24h | | Acute |
| Acute stage: stroke onset within 2 weeks of stroke onset;subacute stage:stroke onset within 2 to 28 days; recovery stage: stroke onset after 28 days.  F,female;M,male;d,days;h,hours;C,control group;T,treatment group;SD, standard deviation; SRs, systematic reviews; RCTs, randomized controlled trial;G1,group 1;G2,group 2. | | | | | | | | | |
| Supplemental Table IX. Characteristics of included SRs and their original RCTs for Chuanxiong-type preparations (continued) | | | | | | | | | |
| **SRs** | **Original RCTs** | **Interventions** | | **Primary outcomes** | | **Follow-up during** | | **Conclusion** | |
|  |  | **Treatment group** | **Control group** |  | |  | |  | |
| Yuan 2008^63^ | Fan 2003^64^ | Chuanxiong 80mg+ RT bid×14d | RT bid×14d | Neurological improvement* | | na | | Positive | |
|  | Wang 2006^65^ | Chuanxiong 150mg + RT qd×14d | RT qd×14d | Neurological improvement*;  FAE^#^ | | na | | Positive | |
| Wu 2007(b)^66^ | Guo 2002^15^ | Chuanxiong 80mg+ RT×14d | RT×14d | Neurological improvement*;  death | | na | | Positive | |
| na, not available; qd, once a day; bid,twice a day;d,days;RT,routine treatment;SRs, systematic reviews; RCTs, randomized controlled trial; FAE, Frequency of adverse events  * Neurological Deficit Score.  ^#^ hemorrage or elevation of liver enzymes. | | | | | | | | | |

| Supplemental Table X. Characteristics of included SRs and their original RCTs for Puerarin | | | | | | | | | | |
| --- | --- | --- | --- | --- | --- | --- | --- | --- | --- | --- |
| **SRs** | **Original RCTs** | **Number of patients** | **Stroke type** | **Time since stroke** | **Stage** | **Interventions** | | **Primary outcomes** | **Follow-up during** | **Conclusion** |
|  |  |  |  |  |  | **Treatment group** | **Control group** |  |  |  |
| Tan 2008^67^ | Chao 2004^68^ | 98 | Ischemic | ＜10d | Acute | Puerarin 200mg iv qd + RT ×15d | RT ×15d | Death/disability | 6 mo | Positive |
| Acute stage: stroke onset within 2 weeks of stroke onset;subacute stage:stroke onset within 2 to 28 days; recovery stage: stroke onset after 28 days.  d,days;mo,months; SRs, systematic reviews; RCTs, randomized controlled trial; RT,routine treatment; qd,once a day. | | | | | | | | | | |

| Supplemental Table XI. Characteristics of included SRs and their original RCTs for Milk vetch | | | | | | | | | | |  |
| --- | --- | --- | --- | --- | --- | --- | --- | --- | --- | --- | --- |
| **SRs** | **Original RCTs** | **Number of patients(G1/G2)** | **Age**  **(yrs)** | | **Gender (M/F)** | **Stroke type** | | **Time since stroke** | | **Stage** |  |
| Wu 2007(b)^66^ | Zhou 2003^69^ | 120(60/60) | T:42-80(mean 61);  C:41-82(mean 59) | | T:32/28;  C:31/29 | Ischemic | | 6h-1wk | | Acute |  |
| Acute stage: stroke onset within 2 weeks of stroke onset;subacute stage:stroke onset within 2 to 28 days; recovery stage: stroke onset after 28 days.  F,female;M,male; h,hours;wk,weeks;C,control group;T,treatment group; SRs, systematic reviews; RCTs, randomized controlled trial;G1,group 1;G2,group 2. | | | | | | | | | | |  |
| Supplemental Table XI. Characteristics of included SRs and their original RCTs for Milk vetch (continued) | | | | | | | | | | | |
| **SRs** | **Original RCTs** | **Interventions** | | | | | **Primary outcomes** | | **Follow-up during** | | **Conclusion** |
|  |  | **Treatment group** | | **Control group** | | |  | |  | |  |
| Wu 2007(b)^66^ | Zhou 2003^69^ | Milk vetch 50ml+ RT×15d | | RT×15d | | | Death | | na | | Positive |
| na, not available; d, days; RT, routine treatment;SRs, systematic reviews; RCTs, randomized controlled trial. | | | | | | | | | | | |

| Supplemental Table XII. Characteristics of included systematic reviews and their original RCTs for Qing Kai Ling | | | | | | | |
| --- | --- | --- | --- | --- | --- | --- | --- |
| **SRs** | **Original RCTs** | **Number of patients(G1/G2)** | **Age**  **(yrs)** | **Gender (M/F)** | **Stroke type** | **Time since stroke** | **Stage** |
| Wu 2007(b)^66^ | Huo 2004^70^ | 128(68/60) | T:46-73(mean 59.5);  C:40-75(mean 59) | T:40/28;  C:38/22 | Ischemic | na | Acute |
|  | Yu 1999^71^ | 40(20/20) | T: mean 54(SD 10);  C: mean 58(SD 10) | T:12/8;  C:13/7 | Ischemic | 1-3d | Acute |
| Acute stage: stroke onset within 2 weeks of stroke onset; subacute stage: stroke onset within 2 to 28 days; recovery stage: stroke onset after 28 days.  na, not available; F, female;M, male; d, days; C, control group;T, treatment group; SRs, systematic reviews; RCTs, randomized controlled trial;G1,group 1;G2,group 2. | | | | | | | |

| Supplemental Table XII Characteristics of included SRs and their original RCTs for Qing Kai Ling (continued) | | | | | | |
| --- | --- | --- | --- | --- | --- | --- |
| **SRs** | **Original RCTs** | **Interventions** | | **Primary outcomes** | **Follow-up during** | **Conclusion** |
|  |  | **Treatment group** | **Control group** |  |  |  |
| Wu 2007(b)^66^ | Huo 2004^70^ | Qing Kai Ling 40ml + RT | RT | Death | na | Positive |
|  | Yu 1999^71^ | Qing Kai Ling 50 ml + RT×14d | RT×14d | Death | 4wk | Positive |
| na, not available; d, days; wk, weeks; RT, routine treatment; SRs, systematic reviews; RCTs, randomized controlled trial. | | | | | | |
